# Mitochondrial integrated stress response activation creates a therapeutic vulnerability to MCL-1 inhibition in acute myeloid leukemia

**DOI:** 10.1038/s41419-026-09037-w

**Published:** 2026-06-25

**Authors:** Lauren Brakefield-Laird, Amit Budhraja, Peter M. Hall, Dixie Brewington, Jamila Moore, Josi Lott, Yonghui Ni, Dennis Voronin, Oliver Grant-Chapman, Tresor O. Mukiza, Tristen Wright, Yong-Dong Wang, Sandi Radko-Juettner, Shondra M. Pruett-Miller, Stanley Pounds, Peter Vogel, Joseph T. Opferman

**Affiliations:** 1 Department of Cell and Molecular Biology, St. Jude Children’s Research Hospital, Memphis, TN USA; 2 Biomedical Sciences Program, University of Tennessee Health Science Center, Memphis, TN USA; 3 The Center for Advanced Genome Engineering, St. Jude Children’s Research Hospital, Memphis, TN USA; 4 Preclinical Therapeutics Program, St. Jude Children’s Research Hospital, Memphis, TN USA; 5 Center of Excellence for Leukemia Studies St. Jude Children’s Research Hospital, Memphis, TN USA; 6 Department of Biostatistics, St. Jude Children’s Research Hospital, Memphis, TN USA; 7 St. Jude Graduate School of Biomedical Sciences, St. Jude Children’s Research Hospital, Memphis, TN USA; 8 Department of Pathology, St. Jude Children’s Research Hospital, Memphis, TN USA; 9 Comparative Pathology Core, St. Jude Children’s Research Hospital, Memphis, TN USA

**Keywords:** Targeted therapies, Kinases

## Abstract

MCL-1 (myeloid cell leukemia-1) promotes survival and confers therapeutic resistance in acute myeloid leukemia (AML), particularly in high-risk subtypes harboring *KMT2A* rearrangements (*KMT2A*-r). Clinical trials involving patients with hematological malignancies treated with MCL-1 inhibitor monotherapy have been hampered by dose-limiting toxicity and poor response rates. Therefore, we sought to identify combinatorial treatment approaches to enhance the efficacy of MCL-1 inhibitors with the goal of improving response rates and limiting toxicities. Here, we report the inhibition of electron transport chain (ETC) complex I (CI) function as a synthetic lethal partner for MCL-1 inhibition. Co-targeting CI and MCL-1 synergistically reduces the viability in AML cell lines and patient-derived xenograft (PDX) samples in vitro, while significantly prolonging survival in mice bearing PDX AML, indicating the preclinical potential for this combinatorial therapy. These findings provide a mechanistic rationale and preclinical evidence for dual inhibition of MCL-1 and CI as a therapeutic strategy, offering a potential path to overcome resistance to single-agent MCL-1 inhibitors and improve outcomes for patients with high-risk AML. Mechanistically, we reveal that CI inhibition induces the activation of the integrated stress response, resulting in ATF4 activation downstream of the eIF2α kinase, HRI (Heme-regulated inhibitor). HRI activation via CI inhibition is dependent on the mitochondrial stress messenger, DELE1. Together, these results indicate that co-inhibition of MCL-1 and ETC CI function has the potential for improving responses in patients with *KMT2A*-r AML.

## Introduction

Acute myeloid leukemia (AML) is an aggressive hematologic malignancy characterized by clonal expansion of myeloid progenitor cells and impaired differentiation [1]. Clinical outcomes vary widely, with certain genetic subtypes, including *KMT2A* (previously known as *MLL*) rearrangements (hereafter referred to as *KMT2A*-r*)*, representing high-risk disease associated with poor prognosis and resistance to standard therapies [1, 2]. The intrinsic pathway of apoptosis is regulated by the BCL-2 family of proteins, where anti-apoptotic members such as BCL-2, BCL-XL, and MCL-1 promote survival, and pro-apoptotic effectors like BAX and BAK drive cell death [3]. Dysregulation of this balance contributes to therapeutic resistance, making BCL-2 family proteins attractive therapeutic targets in cancer. BH3 mimetics are small molecules that mimic the function of pro-apoptotic BH3-only proteins to inhibit anti-apoptotic BCL-2 family members and induce apoptosis in cancer cells [4]. Venetoclax is a selective BCL-2-targeting BH3 mimetic that is approved for use in combination with hypomethylating agents or low-dose cytarabine for the treatment of newly diagnosed AML in patients who are ineligible for intensive chemotherapy [5, 6]. However, resistance often emerges through compensatory upregulation of MCL-1 [7–12]. MCL-1 inhibitors have demonstrated potent activity in preclinical AML models and some have been evaluated in clinical trials; however, their development has been constrained by observed toxicities [13–16].

AML is a heterogeneous disease, not only at the genetic and epigenetic levels but also in its cellular and metabolic composition [17, 18]. Among AML populations, leukemic stem cells (LSCs) display distinct metabolic programs compared with bulk leukemia cells, often relying on mitochondrial oxidative phosphorylation (OXPHOS) rather than anaerobic glycolysis for energy production and survival [19, 20]. In contrast, normal hematopoietic stem cells primarily rely on anaerobic glycolysis to protect from oxidative stress and maintain their stemness, but switch to oxidative metabolism during differentiation and proliferation [21]. The metabolic dependency of LSCs on OXPHOS contributes to their quiescence, resistance to conventional chemotherapies, and ability to drive disease relapse [22, 23]. The metabolic heterogeneity of AML therefore represents both a challenge and an opportunity: by identifying and targeting specific metabolic vulnerabilities in LSCs, such as OXPHOS or mitochondrial stress pathways, it may be possible to selectively eradicate these therapy-resistant cells without harming normal hematopoietic stem cells. Targeting the unique metabolic dependencies of LSCs offers a promising strategy to enhance therapy, prevent relapse, and eradicate AML at its root.

Targeting mitochondrial metabolism, particularly electron transport chain (ETC) complex I (CI), has emerged as a promising strategy in hematologic malignancies, including AML. CI is the first enzyme complex of the mitochondrial ETC and is important for facilitating OXPHOS and ATP production [24]. Inhibiting CI disrupts mitochondrial respiration and induces mitochondrial stress [25]. IACS-010759 (hereafter referred to as IACS) is a potent, selective small-molecule CI inhibitor that has shown preclinical efficacy in AML models [26–28]. Other experimental CI inhibitors are being investigated with similar mechanistic rationales, emphasizing the vulnerability of metabolically dependent cancer cells [29–32]. Clinical evaluation of IACS demonstrated activity in relapsed/refractory AML and other malignancies, although dose-limiting toxicities, including lactic acidosis and neurotoxicity, have limited its use as monotherapy [26]. Preclinical studies suggest that combining CI inhibitors with other therapies has the potential to selectively eradicate LSCs and overcome resistance, highlighting CI inhibition as a promising avenue to exploit AML’s metabolic vulnerabilities and improve long-term treatment outcomes [33–37].

The mitochondrial stress response, particularly the DELE1-HRI-ATF4 axis, is a critical mechanism by which cells sense and adapt to mitochondrial dysfunction. Upon stress, DELE1 can be cleaved, activating the kinase HRI to phosphorylate eIF2α, leading to induction of the transcription factor ATF4 [38, 39]. ATF4 can then induce the expression of genes regulating stress adaptation, metabolism, and apoptosis [40–43]. In mouse models of AML, the repression of ATF4 impairs AML growth, delaying leukemogenesis and reducing leukemia-initiating cell activity [44]. These findings underscore the therapeutic potential of the mitochondrial stress response: targeting DELE1, HRI, or ATF4 could selectively disrupt AML cell adaptation to stress, enhance sensitivity to treatment, and improve outcomes, making this pathway a promising target for novel AML therapies.

In this study, we investigated how metabolic modulation can enhance the efficacy of MCL-1 inhibition in AML. We identified a synthetic lethal interaction between inhibition of mitochondrial CI and MCL-1 blockade. Specifically, the selective CI inhibitor IACS synergized with multiple MCL-1 inhibitors to enhance AML cell death. Mechanistically, this combination activated the DELE1-HRI-ATF4 arm of the integrated stress response (ISR), driving cell death. These results highlight the therapeutic value of co-targeting MCL-1 and mitochondrial metabolism to achieve robust anti-leukemic activity in both in vitro and in vivo models.

## Materials and methods

### Cells and cell culture

MV4-11 cells were acquired from the American Type Culture Collection (ATCC; Manassas, VA, USA). ML2, THP1, OCI-AML3, U937, HL60, CHRF-288-11, M-O7E, and Molm-13 cells were a gift from Charles Mullighan at St. Jude Children’s Research Hospital (SJCRH), Memphis, TN, USA. AML cell lines were cultured in Roswell Park Memorial Institute (RPMI) 1640 media (Thermo Fisher Scientific, Waltham, MA, USA) supplemented with 10% fetal bovine serum (Gibco, Billings, MT, USA), 55 µM 2-mercaptoethanol, 2 mM glutamine, and 1% penicillin-streptomycin solution (Invitrogen, Waltham, MA, USA). M-O7e cells were cultured in the same RPMI formulation described above and supplemented with 10 ng/mL human IL3 (Peprotech, Cranbury, NJ, USA). Cell lines were authenticated via short tandem repeat profiling through the Hartwell Center for Biotechnology (SJCRH). Cas9-expressing MV4-11 cells were generated using lentiCas9-Blast, a gift from Feng Zhang (Addgene viral prep# 52962-LV). A stably expressing single cell clone was generated under blasticidin selection (15 µg/mL). All cell lines were subjected to frequent mycoplasma testing using the MycoAlert Mycoplasma Detection Kit (Lonza, Basel, Switzerland) following the manufacturer’s protocol and were maintained in culture for no more than 6 weeks. S63845 (Cat. No. HY-100741), S64315 (Cat. No. HY-112218), and IACS-010759 (Cat. No. HY-112037) were obtained through MedChem Express (Monmouth Junction, NJ, USA). AMG 176 and AZD5991 compounds were obtained through the Department of Chemical Biology and Therapeutics (SJCRH). Thapsigargin was obtained through Millipore Sigma (Burlington, VT, USA). A-1331852 was obtained through MedKoo Biosciences (Durham, NC, USA), venetoclax (ABT-199) was obtained from LC Laboratories (Woburn, MA, USA), and navitoclax (ABT-263) was obtained from ApexBio Technology LLC. (Houston, TX, USA).

### Ex Vivo patient-derived xenograft isolation and culture

Leukemia cells originating from two pediatric patients (SJAML016500_D1 and SJAML030440_R2) harboring *KMT2A*-r AML were established at SJCRH and can be accessed using the Public Resource of Patient-derived and Expanded Leukemia (PROPEL) repository (https://propel.stjude.cloud/). SJAML016500_D1 was derived from a 12-year-old patient at the time of diagnosis, and SJAML030440_R2 was derived from a 5-year-old patient at relapsed disease and are labeled in figures respectively as “diagnosis” and “relapsed”. For in vivo propagation, 1.5–1.75E6 cells were administered by tail vein injection into immunodeficient Non-Obese Diabetic (NOD) Cg-*Prkdc*^*scid*^ *Il2rg*^*tm1Wjl*^ Tg(CMV-IL3, CSF2, KITLG)1Eav/MloySzJ (NSG-SGM3) 8- to 12-week-old female mice (Jackson Laboratories, Bar Harbor, ME, USA). Retro-orbital bleeds were performed every 4 weeks until a humane endpoint (presence of hind leg weakness/paresis, delayed response to touch, hunched posture, tachypneic) was reached. Disease progression was assessed by flow cytometry using a panel to detect mouse CD45, human CD19, human CD34, human CD33, human CD7, human CD45, and DAPI. Once euthanized, bone marrow and spleen cells were harvested by flushing the femurs or mashing the spleens through a 100 μm filter. Collected cells were centrifuged at 1200 RPM for 5 min at 4 °C and underwent red blood cell lysis using the BD Pharm Lyse lysing reagent (BD Biosciences, Franklin Lakes, NJ, USA) following the manufacturer’s protocol. Mouse cells were depleted using mCD45-biotin antibody (clone 30-F11, Cat No.: 103104, Biolegend San Diego, CA, USA), streptavidin magnetic particles (Roche, Basel, Switzerland), and a Miltenyi magnet system following the manufacturer’s protocol. Ex vivo cells were maintained for up to 24 h in StemSpan Serum-Free Expansion Medium II (StemCell Technologies, Vancouver, BC, Canada) supplemented with 10 ng/mL GCSF, 100 ng/mL SCF, 10 ng/mL IL3, 100 ng/mL IL6, 100 ng/mL TPO, and 100 ng/mL FLT3 ligand (Peprotech).

### CRISPR knockout library design, assembly, and validation

The ISR CRISPR knockout KO library was designed using the CRISPick tool and composed of up to five high-confidence *Streptococcus pyogenes* single-guide RNAs (sgRNAs) per gene [45, 46] by the Center for Advanced Genome Engineering Core Facility (CAGE, SJCRH). Briefly, CRISPick was used to identify the top candidate sgRNAs for each gene, which were then subjected to another round of in-house off-target filtering. Additionally, non-targeting control sgRNAs were incorporated to comprise approximately 10% of the final library composition. Oligos with appropriate overhangs were generated by TWIST Bioscience (South San Francisco, CA, USA). Oligo library amplification and Gibson Assembly into the lentiGuide-Puro vector backbone (Addgene #52963) was performed as previously described [47, 48]. The ISR KO plasmid library underwent assembly, amplification, and validation in the CAGE following the Broad Institute GPP protocol (https://portals.broadinstitute.org/gpp/public/resources/protocols) with the only modification being the use of Endura DUOs electrocompetent cells. The single-end 100-cycle sequencing was performed on a NovaSeq 6000 (Illumina, San Diego, CA, USA) by the Hartwell Center. The Human CRISPR Metabolic Gene KO Library DNA was a gift from David Sabatini (Addgene #110066) and was amplified in the same manner [49]. Validation of both libraries was performed to assess for sgRNA presence and representation using calc_auc_v1.1.py (https://github.com/mhegde/) and count_spacers.py [47]. A list of genes and sgRNA sequences for the ISR KO library is available in Supplementary (S) Table S1.

### CRISPR KO library lentiviral particle production

Lentiviral particles for the Human CRISPR Metabolic KO Library and ISR KO library were manufactured by the Vector Development and Production Laboratory (SJCRH) as previously described [50]. Briefly, 2e6 SJ293TS-DPB cells/mL (500 mL total) were transfected with either the Human CRISPR Metabolic KO or ISR KO library plasmid DNA and the helper plasmids, pCAG-kGP1-1R-AF (gagpol), pCAG-VSVG-AF (VSV-G), and pCMV-RTR2-AF (Rev), at a ratio of 14:4:2:0.25, respectively. Six hours after transfection, cells were diluted in 500 mL with freestyle culture media and treated with Benzonase at a final concentration of 6.25 µ/mL. After 48 h, vector supernatants were harvested, clarified by centrifugation, and filtered through both 0.45- and 0.22-µm filters. The lentiviral-containing supernatant was adjusted to 300 mM NaCl with 50 mM Tris, pH 8.0, before being loaded onto Mustang Q XT5 ion-exchange capsule (Cytiva, Marlborough, MA) per the manufacturer’s instructions using an ÄKTA™ Avant chromatography system (Cytiva). Lentiviral particles were eluted from the column using 2 M NaCl, 50 mM Tris pH 8.0. Eluates from the Mustang Q XT5 ion-exchange capsule were diluted fivefold with phosphate-buffered saline (PBS) and diafiltrated twice in PBS using a Vivaflow 50 with a 100,000 molecular weight cutoff (Sartorius AG, Goettingen, Germany) according to the manufacturer’s instructions. The final diafiltration was performed in X-VIVO 15 media (Lonza). The purified and concentrated vectors were passed through a 0.22-µm filter and stored at −80 °C until use. To determine viral titers, purified/concentrated vector preps were titered on HOS-6A5 cells. Transduced HOS6A5 cells were harvested 4 days post transduction. Droplet digital PCR was used to determine the viral titer as follows: genomic DNA was isolated from transduced cells, digested with MspI, and used as a template in PCR with a probe against the HIV Psi element of the vector and a probe against human RPP30, and endogenous control was assumed to be ~2 copies/cell. The primer-probe sets listed in Table S2 were used to amplify the endogenous control gene, *RPP30* and the HIV psi sequence.

### CRISPR KO screens

The Human CRISPR Metabolic Gene KO Library was a gift from David Sabatini (Addgene #110066) [49]. MV4-11 cells were transduced with the Human CRISPR Metabolic Gene KO Library at a multiplicity of infection (MOI) of 0.5 with a sgRNA coverage of 1000x. Twenty-four hours after the transduction, 2 µg/mL puromycin was added for selection and kept in the media for 7 d before removal. Cells were then cultured in media supplemented with 10 nM S64315, 350 nM AMG 176, or volume-matched DMSO for another 24 h, followed by culture in drug-free media for up to four population doublings after drug treatment. MV4-11 Cas9 cells were transduced with the ISR KO library at an MOI of 0.5 and a sgRNA coverage of 5000x. Twenty-four hours after transduction, puromycin was added for selection in the same manner as described above. Cells were cultured in media containing DMSO or a combination of 4 nM S64315 and 160 nM IACS. The drugs were washed from the media after 24 h of exposure, and the cells were cultured for 13 population doublings in drug-free media. For both screens, genomic DNA was extracted from the surviving cells using the DNeasy Blood and Tissue Kit (Qiagen, Hilden, Germany) following the manufacturer’s instructions, then amplified with PCR before sgRNA sequencing via Nextseq (Illumina). The MAGeCK-VISPR algorithm was used to compare the drug-treated samples to the DMSO-treated samples [51]. For the metabolic CRISPR KO screen, the top hits with false discovery rates <30% were put into Enrichr for pathway analyzes [52, 53]. For the targeted ISR screen, robust rank aggregation (RRA) was used to identify the top hits.

### KO cell line creation

KOs within MV4-11 and THP1 cell pools were generated using CRISPR-Cas9 technology by the CAGE. Two million cells were transiently transfected with precomplexed ribonuclear protein containing 300 pmol of each chemically modified sgRNA (Synthego, Redwood City, CA, USA) and 100 pmol 3X NLS SpCas9 protein (SJCRH Protein Production Core) via nucleofection (Lonza 4D-Nucleofector™ X-unit) using solution SF and program DJ-100 in a 100-µL cuvette according to the manufacturer’s protocol. Three days after nucleofection, cell pellets with approximately 100,000 cells were lysed, and gene-specific amplicons were generated and then sequenced by targeted next-generation sequencing (NGS) as previously described [54]. NGS data were processed using CRIS.py [55]. After expansion, the cell pools were authenticated using the Power Plex® Fusion System (Promega, Madison, WI, USA) by the Hartwell Center and tested for mycoplasma using the MycoAlert^TM^Plus Mycoplasma Detection Kit (Lonza). Highly enriched KO pools were serially diluted to achieve up to three enriched KO cell lines per genotype. The lines were further verified by Western blot and NGS. Editing construct and NGS primer sequences are listed in Table S3.

### Immunoblotting and antibodies

Protein was extracted by resuspension of cell pellets in RIPA buffer supplemented with final concentrations of 10 mM sodium fluoride (MilliporeSigma, Burlington, MA, USA), 1 mM sodium orthovanadate (MP Biomedicals, Santa Ana, CA, USA), 1x protease inhibitor (MilliporeSigma), and 1 mM phenylmethylsulfonylfluoride (Thermo Fisher Scientific). Gels were loaded with 30 to 40 μg of protein, run on NuPage 4% to 12% bis-tris gradient gels (Invitrogen), and transferred to Immobilon PVDF membranes (MilliporeSigma). The antibodies used were anti-ATF4 (Cl. D4B8, Cat. No.: 11815S), anti-BAK (Cat. No.: 3814S), anti-BAX (Cat. No.: 2772S), anti-EIF2α (Cat. No.: 9722S), and anti-phospho-EIF2α (Ser51, Cat. No.:9721S) from Cell Signaling Technologies (Danvers, MA, USA); anti-HRI (EIF2AK1, Cat. No.: 20499-1-AP) from Proteintech (Rosemont, IL, USA); and anti-Actin (Cl. C4. Cat. No.: MAB1501) from MilliporeSigma. Anti-rabbit and anti-mouse horseradish peroxidase-conjugated secondary antibodies were obtained from Invitrogen. Immunoblots were developed using Odyssey Fc from LI-COR Biosciences (Lincoln, NE, USA), and Image Studio v6.0.0.28 was used to produce the Western blot figures. All experiments are representative of three or more independently performed experiments. Uncropped Western blots available as Fig. S15.

### Cell death experiments

Cells were seeded in 96-well plates between 50,000 and 100,000 cells per well and treated with the indicated drugs (IACS, AMG 176, AZD5991, S63845, S64315, A-1331852, venetoclax, or navitoclax solubilized in DMSO, or DMSO–vehicle control) for 24 h unless otherwise indicated. For the generation of dose response curves, the cells were treated with BH3 mimetics over a concentration range of 40 µM to 40 nM in twofold serial dilutions. For combination treatments, IACS was combined with the MCL-1 inhibitors at the concentrations indicated on the figures. All cell death experiments were normalized to a DMSO-treated control by setting DMSO-treated cell viability to 100%. Cell death was assessed by flow cytometry using a BD FACS Canto II (BD Biosciences), and data were analyzed using FlowJo Software (v10.10, BD Biosciences). For cell death experiments, APC Annexin V (AV; Biolegend) was used at a concentration of 1:100. Propidium iodide (PI; MilliporeSigma) was used at a final concentration of 0.5 μg/mL. Cell viability data were determined and reported using AV and propidium iodide double-negative cells as previously described [56].

### Quantification of drug synergy

The drug combination responses were calculated based on the Bliss reference model using SynergyFinder+ [57]. The SynergyFinder+ online software and R package were used to generate Bliss scores with *p*-values from cell viability data. According to the SynergyFinder+ paper, the Bliss *p*-value is “an empirical *p*-value to assess the significance of the difference between the estimated average synergy score over the whole dose matrix and the expected synergy score of zero under the null hypothesis of non-interaction” [57]. Scores are considered synergistic if >10 and additive if between −10 and 10. To determine statistical significance between wild-type (WT) and KO genotypes, we obtained the Bliss score and Bliss *p*-value, then converted the Bliss *p*-values into *Z*-scores to calculate the standard deviation of bootstrapping. Finally, we applied a *Z*-test to compare the difference between the two Bliss scores and obtain a *Zp*-value. A *Zp*-value less than 0.05 was considered statistically significant.

### ATP production measurements with the Seahorse Assay

ATP production was measured using the Seahorse XF Mito Stress Test (Agilent, Santa Clara, CA, USA) and was performed following the manufacturer’s protocol on a Seahorse XFe96 Analyzer. All cell lines and PDX cells were treated with DMSO or 50 nM IACS in the culture media for 1 h. Cell lines were treated with the S64315 doses indicated on the graphs for 24 h. Cells were plated at a density of 100,000 cells per well into 96-well Seahorse plates that were precoated with 12 μg/mL Cell Tak (Thermo Fisher Scientific). Before plating, culture media were exchanged for Seahorse XF RPMI Medium supplemented with 1 mM sodium pyruvate, 2 mM L-glutamine and 10 mM glucose. IACS was kept in the media for the duration of the assay. Cells were placed in a CO_2_-free incubator to outgas for 1 h. The Mito Stress Test was performed using a final concentration of 1.5 µM oligomycin (Thermo Fisher Scientific), 2 µM carbonyl cyanide 4-(trifluoromethoxy) phenylhydrazone (FCCP; Millipore Sigma), and 0.5 µM rotenone/antimycin A (Sigma Life Sciences/Thermo Fisher Scientific). For each biological replicate, three or more technical replicates were performed. The total cell number per well was obtained through fluorescent cell counting of cells stained with 15 μM Hoechst 33342 (Thermo Fisher Scientific) on Cytation 5 (v 1.0.0-694; BioTek, Winooski, VT, USA). Data was normalized to total cell number and analyzed using Agilent Seahorse Analytics online software v1.0.0-694. Mitochondrial ATP (mitoATP) and glycolytic ATP (glycoATP) production rates were calculated using the Agilent Seahorse Analytics online software, where oxygen consumption rates (OCR) and extracellular acidification rates (ECAR) values were converted to mitoATP and glycoATP as previously described [58]. Briefly, glycoATP was estimated from ECAR values that were converted to proton efflux rates, and the mitochondrial contribution to acidification was subtracted to calculate glycoATP production [59]. Mitochondrial ATP production (mitoATP) was estimated from the OCR that is coupled to ATP production during OXPHOS by subtracting the OCR remaining after oligomycin treatment from the basal OCR and then converted to ATP production using a conversion factor [59]. Graphs were made in GraphPad Prism (v10.5.0). Each experiment was performed *n*=three or more times. Graphs presented in the figures depict representative experiments.

### BH3 profiling

Dynamic BH3 profiling was conducted using a previously published method [60]. Briefly, 500,000 cells were seeded and treated with either DMSO or 50 nM IACS for 24 h. Cells were collected, washed with PBS and stained with AquaZombie viability dye (Biolegend). Cells were exposed to a 0.0001% digitonin (Thermo Fisher Scientific) for membrane permeabilization and then exposed to concentrations increasing up to 5 μM of human BIM peptide (sequence: MRPEIWIAQELRRIGDEFNA) for 1 h at 23 °C. After BIM peptide exposure, 4% formaldehyde in PBS (Thermo Fisher Scientific) was added to terminate the peptide exposure, followed by neutralization with N2 buffer (1.7 M Tris base, 1.25 M glycine, pH 9.1). The cells were stained with 1:40 with anti-cytochrome *c* antibody (clone 6H2.B4, Biolegend) overnight. The next day, cells were washed twice with 2% FBS in PBS and Cytochrome *c* (Cyt *c*) release was assessed by flow cytometry using previously described gating strategies [61].

### RNA sequencing

Five million AML cells were plated and treated with DMSO or 50 nM IACS for 8 or 24 h for three independent replicates. Total RNA was isolated using the RNeasy kit (Qiagen) following the manufacturer’s protocol with the on-column DNA digest step included. RNA sequencing libraries for each sample were prepared with 1 μg total RNA by using the Illumina TruSeq RNA Sample Prep v2 Kit per the manufacturer’s instructions, and sequencing was completed on the Illumina NovaSeq 6000. The 100-bp paired-end reads were trimmed, filtered against quality controls (Phred-like Q20 or greater) and length (50 bp or longer), and aligned to the human reference sequence GRCh38 (hg38) using CLC Genomics Workbench v20 (Qiagen). For gene expression comparisons, the transcript per million counts were obtained from the CLC RNA-Seq Analysis tool. Differential gene expression analysis was performed with a non-parametric ANOVA followed by Kruskal–Wallis and Dunn’s *post hoc* tests on log-transformed transcript per million counts between three replicates of experimental groups, implemented in Partek Genomics Suite v7.0 (Partek Inc., San Diego, CA, USA). The significantly differentially expressed genes were identified with *p*-value < 0.05 and |log2R| > 0.585 (half-fold change).

### In vivo animal studies

For in vivo combination therapy studies, 8- to 12-week-old, female NSG-SGM3 mice were used and housed under a 12 h–12 h light–dark cycle (light on at 06:00 and off at 18:00), with food and water provided *ad libitum*. Animals were housed in the facility at 20 °C–22 °C with humidity levels maintained at 30–70% at the cage level. All procedures were conducted in compliance with protocols approved by the Institutional Animal Care and Use Committee (IACUC) of SJCRH. Mice were injected with 200,000 or 500,000 PDX cells by tail vein injection. Beginning 5 weeks after injection, peripheral blood was collected weekly from mice via retro-orbital bleeding to assess the presence of human CD45⁺ cells. The Center of Excellence for Leukemia Studies Flow Cytometry Core (SJCRH) collected flow data using the BD FACSymphony A5 (BD Biosciences) to identify and determine the percentage of human CD45^+^ cells as previously described [62].

Mice meeting the enrollment criteria of ~0.5% human CD45 positive (hCD45^+^) cells in the peripheral blood were randomized into treatment arms using a blocked randomization list available from: (https://www.sealedenvelope.com/simple-randomiser/v1/lists). No blinding was performed after randomization. Mice were treated with vehicles, 5 mg/kg IACS PO and vehicle for S64315 IP, 6.5 mg/kg S64315 IP and vehicle for IACS PO, or a combination of 5 mg/kg IACS + 6.5 mg/kg S64315 once daily, for 5 days on and 2 days off for 2 weeks. Two days after the final drug dose, 3 mice per treatment group were euthanized to assess tumor burden at the end of treatment. The remaining mice were monitored for up to 2 months post treatment or until a humane endpoint (presence of hind leg weakness/paresis, delayed response to touch, hunched posture, tachypneic) was reached for survival analysis. For the survival analysis, the baseline was defined as the start date of treatment. The treatment period lasted 12 days (5 days on, 2 days off, and 5 days on). Mice that reached a humane endpoint and did not receive all drug doses were excluded from the analysis for a total of 17 mice (5 from the vehicle-treated group, 5 from the IACS-treated group, 4 from the S64315-treated group, and 3 from the combination-treated group). A total of 54 mice were included in the survival analysis (11 from the vehicle-treated group, 13 from the IACS-treated group, 14 from the S64315-treated group, and 16 from the combination-treated group).

### In vivo formulations

The Analytical Technology Center-Preclinical Formulation Team provided the preclinical formulations for in vivo studies: S64315 free-base was formulated in 2% v/v Kolliphor EL in PBS. IACS free-base was formulated in 0.5% methylcellulose (type 400 cPs) in UP water. Both formulations were stable for 2 weeks at room temperature and at 4 °C at the prepared concentrations.

### Blood serum lactate collections

Blood samples were collected by retro-orbitally and immediately placed in sodium fluoride/potassium oxalate solution at a 1:10 ratio before being placed on ice. Plasma was separated within 15 min of collection by centrifugation at 5600 RPM for 10 min at 4 °C. The resulting serum was placed in a separate tube and stored at 4 °C overnight. Samples were analyzed using the Point Lactate Reagent Set (Canton, MI, USA) according to the manufacturer’s instructions.

### Data analysis, representation, and statistics

IC_50_ values for single-agent curves were calculated using nonlinear regression in GraphPad Prism, using a log(inhibitor) vs normalized response model. Flow cytometry data were analyzed using FlowJo. Seahorse data were processed using the online software Agilent Seahorse Analytics (v1.0.0). For survival analysis, the difference in the treatment effect appears more pronounced during the later stages of follow-up, so a weighted log-rank test (Fleming–Harrington test) that emphasizes differences in survival occurring later in time was applied. We used the Fleming–Harrington test for right-censored data based on counting processes [63], setting the parameters *ρ* = 0 and *λ* = 1 in the weighting function w(t)=[*S*(*t*)]*ρ*[1−*S*(*t*)]*λ*, where *S*(*t*) denotes the Kaplan–Meier estimate of survival at time *t*. The R package “FHtest” was used for this calculation [64]. The resulting *p*-values from the weighted log-rank test are reported in Fig. 5. GraphPad Prism was used for statistical analysis and data visualization. Schematic figures were generated using Biorender. CRISPR knockout screens were conducted using a single biological replicate. All other experiments were performed with at least three biological replicates, unless otherwise specified, and the number of samples (n) is indicated in the figure legends. Outliers were excluded if they deviated markedly from the overall data distribution or were identified using the GraphPad Prism outlier calculator (Grubbs’ test, *α* = 0.5) prior to statistical analysis. Statistical tests are stated in the figure legends. Statistical significance is represented as follows: *P*-values or *Zp*-values ${\boldsymbol{\le }}$ 0.05 are scored as *, ${\boldsymbol{\le }}\,$0.01 are scored as **, ${\boldsymbol{\le }}$ 0.001 are scored as ***, and ${\boldsymbol{\le }}$0.0001 are scored as ****. Data are presented as mean values, with error bars representing the standard error of mean (SEM), unless otherwise indicated. Final figures were made in Adobe Illustrator 2025 (v25.5.1).

## Results

### *KMT2A*-r AML are sensitive to MCL-1 inhibition

To identify a molecular subtype of AML that would benefit from MCL-1 inhibitors, a panel of AML cell lines was treated with increasing doses of various BH3 mimetics to determine the sensitivity of each cell line. The panel includes both pediatric- and adult-derived AML cell lines, different French-American-British classifications, different cytogenetics [65], and different expression of anti-apoptotic BCL-2 family members (Table 1, and Fig. S1). The AML cell lines were treated with either a BCL-XL inhibitor (A-1331852), MCL-1 inhibitor (S63845), a pan BCL-2, BCL-XL, and BCL-W inhibitor (ABT-263, also known as navitoclax), or a BCL-2 inhibitor (ABT-199, also known as venetoclax) for 24 h. Cell death was then assessed by AV and PI-negative cells using flow cytometry. Drug-treated cells were normalized to DMSO-treated cells to obtain cell viability. Five AML cell lines, including three of the four *KMT2A*-r cell lines (ML2, MV4-11, and THP1), were the most sensitive to the tool compound MCL-1 inhibitor, S63845 (Figs. S2A–E and S3, and Table 1). In contrast, other AML cell lines responded preferentially to inhibiting BCL-2 (HL60) or BCL-XL (CHRF 288-11), with several cell lines responding preferentially to navitoclax (M-O7E and Molm-13) (Fig. S2F–I and S3, and Table 1). All cell lines tested exhibited sensitivity to high doses of each BH3 mimetic, suggesting the possibility of toxic off-target effects of the drugs. *KMT2A*-r occurs in up to 60% of infant patients and 15% of pediatric patients with AML and generally reflects an intermediate to poor prognosis [66–68]. Therefore, we decided to optimize a combination therapy to potentiate cell death responses of *KMT2A*-r AML induced by MCL-1 inhibition.

**Table 1:** Characterization of sensitivity to BH3 mimetics in acute myeloid leukemia cell lines.

Cell line	FAB	Age (y), Sex	Mutations	Reference	A-1331852	S63845	ABT-263	ABT-199
HL60	M2	36, F	TP53, CDKN2A, NRAS	PMID 2721272	12790	172.2	5724	63.37
ML2	M4	26, M	KMT2A-AFDN fusion, NRAS, AFDN-HGNC fusion	PMID 6091813	19099	299.8	4873	1636
OCI AML3	M4	57, M	DNMT3A, NRAS, NPM1	PMID 2538684 and 16079892	106726	424.1	14882	22480
Molm-13	M5a	20, M	KMT2A-MLLT3 fusion, FLT3-ITD	PMID 9305600	91059	56614	27424	30712
MV4-11	M5	10, M	KMT2A-AFF1 fusion, FLT3-ITD	PMID 3496132	10901	16.85	457.6	235
THP1	M5	1, M	CSNK2A1-DDX39B fusion, KMT2a, NRAS, TP53	PMID 60970727	27532	128.3	3282	2796
U937	M5	37, M	PICALM-MLLT10 fusion, PTPN11, PTEN, TP53, WT1	PMID 178611	22470	609.3	13441	10757
M-O7E	M7	6m, F	ANO7-DHDH fusion, ETO2-GLIS2 fusion, NRAS	PMID 3261598	27783	10635	2512	14128
CHRF 288-11	M7	1.5, M	NUP98-KDM5 fusion	PMID 2310825	61.94	26108	2847	27089

IC_50_ values in nM of the cell lines treated with 40 μM to 40 nM of BH3 mimetics for 24 h. The BH3 mimetics used include A-1331852 (BCL-XL inhibitor), S63845 (MCL-1 inhibitor, tool compound for S64315), ABT-263/navitoclax (pan BCL-2, BCL-XL, and W inhibitor), and ABT-199/venetoclax (BCL-2 inhibitor). Data is representative of three more independent experiments. IC_50_ values are calculated using nonlinear regression in GraphPad Prism, fitting the data to the log(inhibitor) vs normalized response model.

### Metabolic CRISPR screen reveals CI loss enhances sensitivity to MCL-1 inhibition

**Fig. 1:**
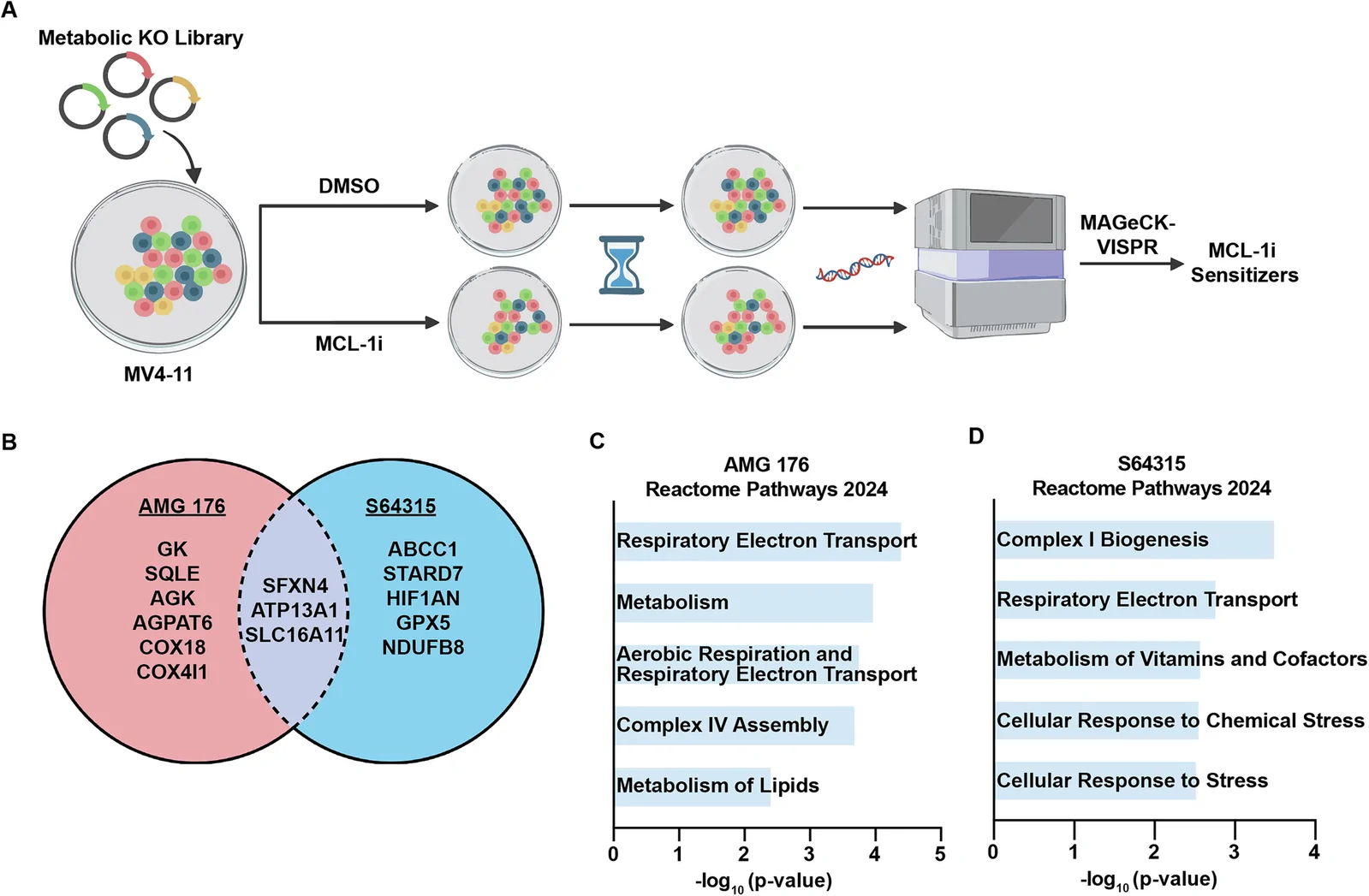
Metabolic CRISPR knockout screen identifies OXPHOS as a sensitizer to MCL-1 inhibition in AML. **A** MV4-11 cells containing the Metabolic CRISPR knockout library were treated with either DMSO, 350 nM AMG 176, or 10 nM S64315for 24 h. Drug-containing media was removed and cells were washed and cultured for up to 4 population doublings in drug-free media. Illumina sequencing was performed on genomic DNA extracted from the DMSO- or MCL-1 inhibitor-treated cells to determine genes that could be targeted to sensitize AML cells to MCL-1 inhibition. **B** Top hits obtained from MAGeCK-VISPR analysis used to identify the loss of the genes that resulted in sensitivity to MCL-1 inhibition when compared to the DMSO control. The listed hits from the screen have a false discovery rate of 30% or less. Enrichr analysis of the pathways identified from the tops hits in **B** from AMG 176 (**C**) or S64315 (**D**) treated cells compared to DMSO.

Given the metabolic heterogeneity of AML, we sought to identify metabolic pathways whose loss would sensitize *KMT2A*-r AML to MCL-1 inhibition. The MV4-11 cell line was chosen as it is sensitive to MCL-1 inhibition and harbors both *KMT2A*-r and FLT3-ITD mutations, which are known to confer poor prognosis (Table 1). MV4-11 cells were transduced with a human metabolic CRISPR KO library to selectively ablate genes involved in cellular metabolism [49] and treated with doses of the MCL-1 inhibitors AMG 176 or S64315 (also known as MIK665), which have been evaluated in recent clinical trials (NCT05209152, NCT02675452, NCT04629443 and NCT03672695). Doses of the inhibitors were chosen that induced approximately 25% cell death at 48 h (Fig. S4A, B). After 4 population doublings, genomic DNA was isolated, sequenced, and analyzed by MAGeCK-VISPR to identify significant hits with a false discovery rate of <30% (Fig. 1A, B). Enrichr analyzes were performed on the significant hits from each inhibitor (Fig. 1C, D). As expected, due to the library, all hits involved metabolic pathways, but several genes were identified that, when lost, conferred sensitivity to MCL-1 inhibitors (Fig. 1B). *SFXN4*, which has been identified as a CI assembly factor, was identified as a common sensitizer amongst both MCL-1 inhibitors [69], along with *ATP13A1* and *SLC16A11*. Furthermore, the genetic loss of the CI subunit *NDFUB8* sensitized the MV4-11 cells to MCL-1 inhibition by S64315. Loss of Complex IV subunits, *COX18* and *COX4I1*, sensitized the MV4-11 cells to AMG 176. Enrichr analyzes of the top hits from either MCL-1 inhibitor revealed loss of complex I and IV components sensitized AML to MCL-1 inhibition (Fig. 1C, D). Furthermore, genes that when lost conferred sensitivity to AMG 176 treatment were also involved in lipid metabolism, such as the lipid kinase *AGK* and the lipid/triglyceride biosynthesis enzyme *AGPAT6* (Fig. 1C). The identification of lipid metabolism loss as a synthetic lethal interaction with MCL-1 inhibition is intriguing, given that MCL-1 has been shown to play an essential role in fatty acid β-oxidation in both normal and malignant contexts [70–72].

### MCL-1 inhibitors synergize with the CI Inhibitor, IACS, to induce apoptosis

**Fig. 2:**
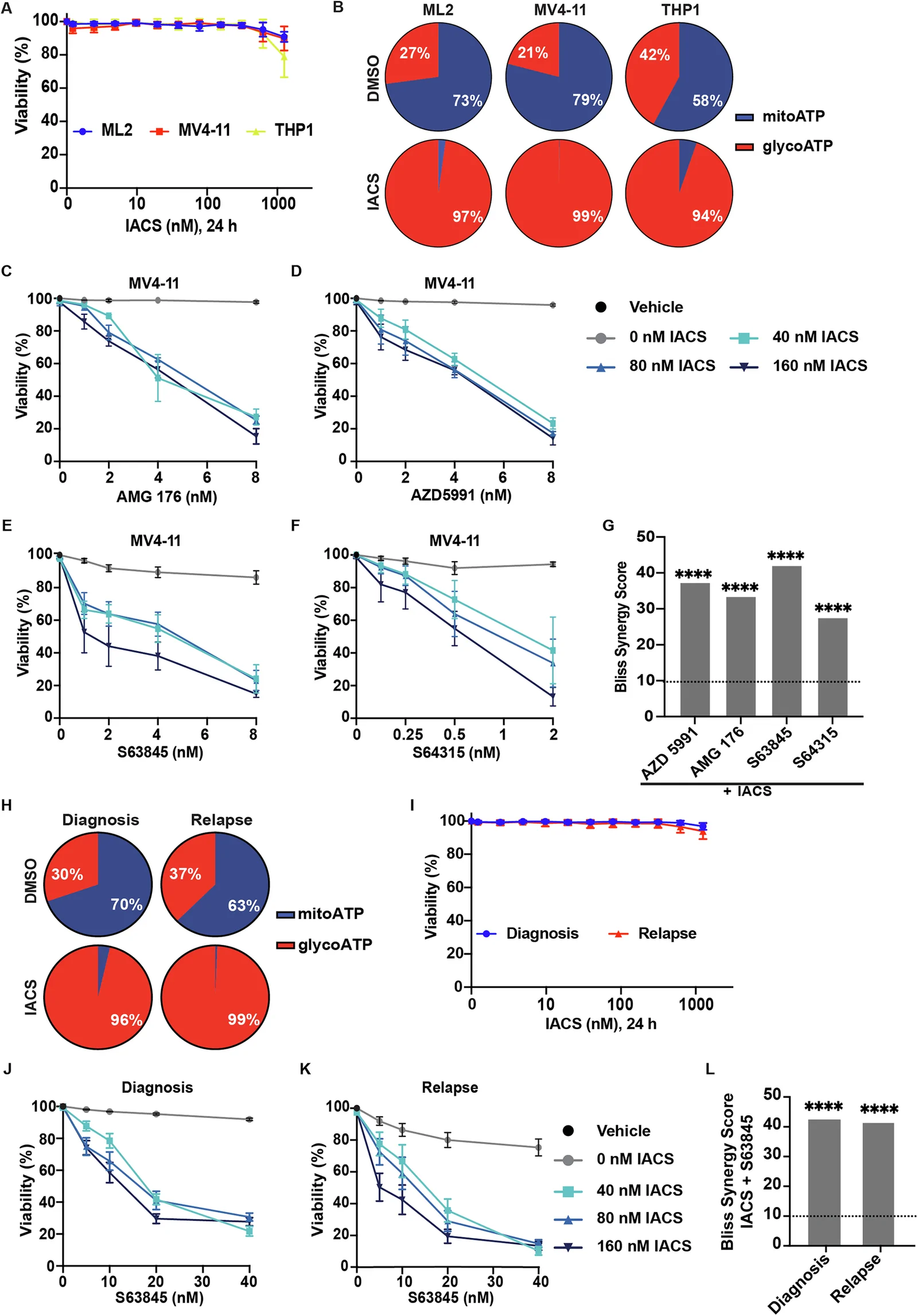
MCL-1 inhibitors synergize with the complex I inhibitor, IACS-010759. **A** Cell viability (AV^-^/PI^-^) of acute myeloid leukemia (AML) cell lines treated with the indicated doses of IACS-010759 (IACS) for 24 h. Cell viability is normalized to the vehicle-treated (DMSO) control, defined as 100%. Graphs represent data from *n* = 3 or more independent experiments, and error bars represent the standard deviation (SD). Data are plotted on a logarithmic scale with the drug concentration shown in nM. **B** Calculated ATP production of ML2, MV4-11, and THP1 cells treated with DMSO or 50 nM IACS for 1 h before undergoing the Seahorse Mito Stress Test to determine the estimated ATP production from OXPHOS (mitoATP) and glycolysis (glycoATP). Values represent calculated estimates of ATP production, not direct ATP measurements. Graphs are representative of three or more experiments. Cell viability (AV^-^/PI^-^) of the MV4-11 cell line treated with the indicated cells doses of IACS combined with AMG 176 (**C**), AZD5991 (**D**), S63845 (tool compound for S64315) (**E**), or S64315 (**F**) for 24 h. Cell viability is normalized to the vehicle-treated (DMSO) control defined as 100%. Graphs represent data from three or more independent experiments and error bars represent the SEM. **G** Bliss synergy scores derived from the data presented in **C**–**F**. The dashed line indicates values over 10 as synergistic. *P*-values associated with Bliss synergy scores are calculated through SynergyFinder+ software. *P*-values ≤ 0.0001 are indicated by ****. Bliss scores were calculated from experiments that were performed with 3 or more replicates. **H** Calculated ATP production of patient-derived xenografts (PDX) cells derived from two AML patients at disease diagnosis or relapse and treated ex vivo with 50 nM IACS or DMSO for 1 h before undergoing the Seahorse Mito Stress Test to determine the estimated ATP production from OXPHOS (mitoATP) and glycolysis (glycoATP). Values represent calculated estimates of ATP production, not direct ATP measurements. Data are represented by *n* = 4 mice per group. **I** Cell viability (AV^-^/PI^-^) of PDX cells treated with the indicated doses of IACS for 24 h. Cell viability is normalized to the vehicle-treated (DMSO) control, defined as 100%. Graphs represent data from *n* = 4 mice and error bars represent the SD. Data are plotted on a logarithmic scale with the drug concentration shown in nM. Cell viability (AV^−^/PI^−^) of PDXs derived from two AML patients at disease diagnosis (**J**) or relapse (**K**) and treated ex vivo with the indicated doses of IACS and S63845 (tool compound for S64315) for 24 h. Cell viability is normalized to the vehicle-treated (DMSO) control defined as 100%. Data are represented by *n* = 4 mice per group and error bars represent the SEM. **L** Bliss synergy scores of the diagnosis or relapse PDX cells treated ex vivo with combined doses of S63845 and IACS. Bliss scores are calculated from experiments using four mice. The dashed line indicates values over 10 as synergistic. *P*-values associated with Bliss synergy scores are calculated through SynergyFinder+ software. *P*-values ≤ 0.0001 are indicated by ****.

Due to the lack of clinically viable complex IV inhibitors, we sought to explore the hypothesis that inhibition of CI function potentiates the ability of MCL-1 inhibitors to induce cell death in AML cell lines. To pharmacologically validate the findings that suggest co-targeting MCL-1 and OXPHOS induces synthetic lethality, we employed IACS as a CI inhibitor that selectively binds to the ND1 subunit of CI and represses OXPHOS [27, 73]. IACS has been tested in clinical trials in both heme and solid cancers as a single agent, exhibiting modest anti-tumor activity at tolerated doses [26]. All three *KMT2A*-r AML cell lines were resistant to IACS-induced cell death, with only minimal cytotoxicity observed at the highest concentrations after 24 h (Fig. 2A). The Seahorse Mito Stress Test was used to directly measure OCR and ECAR in real time. These measurements were used to calculate ATP production derived from OXPHOS (mitoATP) or from glycolysis (glycoATP). Importantly, these values represent calculated estimates of ATP production rather than direct measurements of ATP levels and will be referred to as the calculated values. AML cells treated with 50 nM IACS for 1 h before the Mito Stress Test revealed repressed OXPHOS, as shown by decreased production of calculated mitoATP and forced the cells to almost exclusively produce ATP by glycolysis (Figs. 2B and S5A–C). We also tested the effects of low-dose S64315, as MCL-1 inhibition has been reported to repress cellular respiration [70, 71, 74]. Cells were treated with doses of S64315 for 24 h that induced less than 20% cell death (Fig. S6A), and the respiration was assessed by the Seahorse Mito Stress Test. Though repression of OCR was observed in most of the cell lines, the reduction in OCR was not as robust as what had been observed in IACS-treated cells (Figs. 2B, S6B–E and S5A–C). Furthermore, the repression of OCR was at least partially independent of apoptosis induction, as MV4-11 cells lacking both the apoptotic effectors BAK and BAX (hereafter referred to as DKOs) had a modest reduction in OCR after treatment with S64315 (Fig. S6C). The calculated mitoATP production was minimally, if at all, impacted by single-agent S64315 treatment (Fig. S6F–I). These results indicate that IACS treatment can rapidly inhibit OXPHOS without inducing cell death as a single agent, while inhibition of MCL-1 using S64315 only modestly represses OXPHOS.

To assess whether repression of OXPHOS induced by IACS could potentiate the action of MCL-1 inhibitors, the MV4-11 cell line was treated with four different MCL-1 inhibitors (AMG 176, AZD5991, S63845 [tool compound] or S64315 [next-generation clinical version of S63845]) with or without IACS (Fig. 2C–F). While minimal cell death was observed in cells treated with MCL-1 inhibitors as single agents, the combination treatments significantly reduced cell viability after 24 h of co-treatment. We observed similar effects in the other *KMT2A*-r AML cell lines when treated with the same combinations (Figs. S7A–D and S7F–I). To assess whether the effect of the combinations was synergistic, the combination drug responses were quantified using the Bliss synergy model, where values > 10 indicate a synergistic drug interaction [57]. In all three *KMT2A*-r AML cell lines, we observed a significant synergistic interaction when any of the MCL-1 inhibitors were combined with IACS (Figs. 2G and S7E, J).

To expand our analyzes beyond AML cell lines, *KMT2A*-r PDXs that originated from two patients at either a diagnosis or relapsed disease state were expanded in NSG-SGM3 mice and isolated from the bone marrow for treatment ex vivo. Seahorse Mito Stress Test analyzes of both *KMT2A*-r PDX samples showed that 1 h of pretreatment with 50 nM of IACS decreased the OCR and the calculated mitoATP production, indicating a strong repression of OXPHOS (Fig. 2H and S5D). While cell death was not observed in PDX cells treated with IACS as a single agent after 24 h (Fig. 2I), the combined effects of co-treatment with IACS and S63845 (the tool compound for S64315) potentiated cell death (Fig. 2J, K). Combining IACS with the MCL-1 inhibitor resulted in significantly synergistic killing as determined by Bliss analysis (Fig. 2L). Taken together, these results validate findings of the CRISPR screen, indicating that the repression of CI of the ETC renders *KMT2A*-r AML models more susceptible to cell death by MCL-1 inhibition.

MCL-1 inhibitors trigger intrinsic apoptosis by inducing a BAX- and BAK-dependent mitochondrial outer membrane permeabilization (MOMP) [75]. To test if the cell death observed in response to the combination of IACS and MCL-1 inhibitors was apoptotic, MV4-11 DKO cells were generated by CRISPR gene editing (Fig. S8A). To assess whether the loss of BAX and BAK compromised the ability of IACS to repress mitochondrial ATP production, WT and DKO MV4-11 cells were pretreated with 50 nM IACS for 1 h before assessing mitochondrial function by Seahorse Mito Stress Test. IACS treatment decreased the OCR and the calculated mitoATP production similarly in both the WT and DKO MV4-11 cells (Fig. S8B, C). Furthermore, when DKO cells were treated with IACS combined with the MCL-1 inhibitors S64315, AMG 176, or AZD5991, they were highly resistant to cell death (Fig. S8D–F). These data demonstrate that the pro-apoptotic effectors BAK and BAX are required to mediate cell death in response to the combination treatment, indicating the canonical apoptosis pathway is engaged by the combination therapy.

### IACS primes the mitochondria for apoptosis

**Fig. 3:**
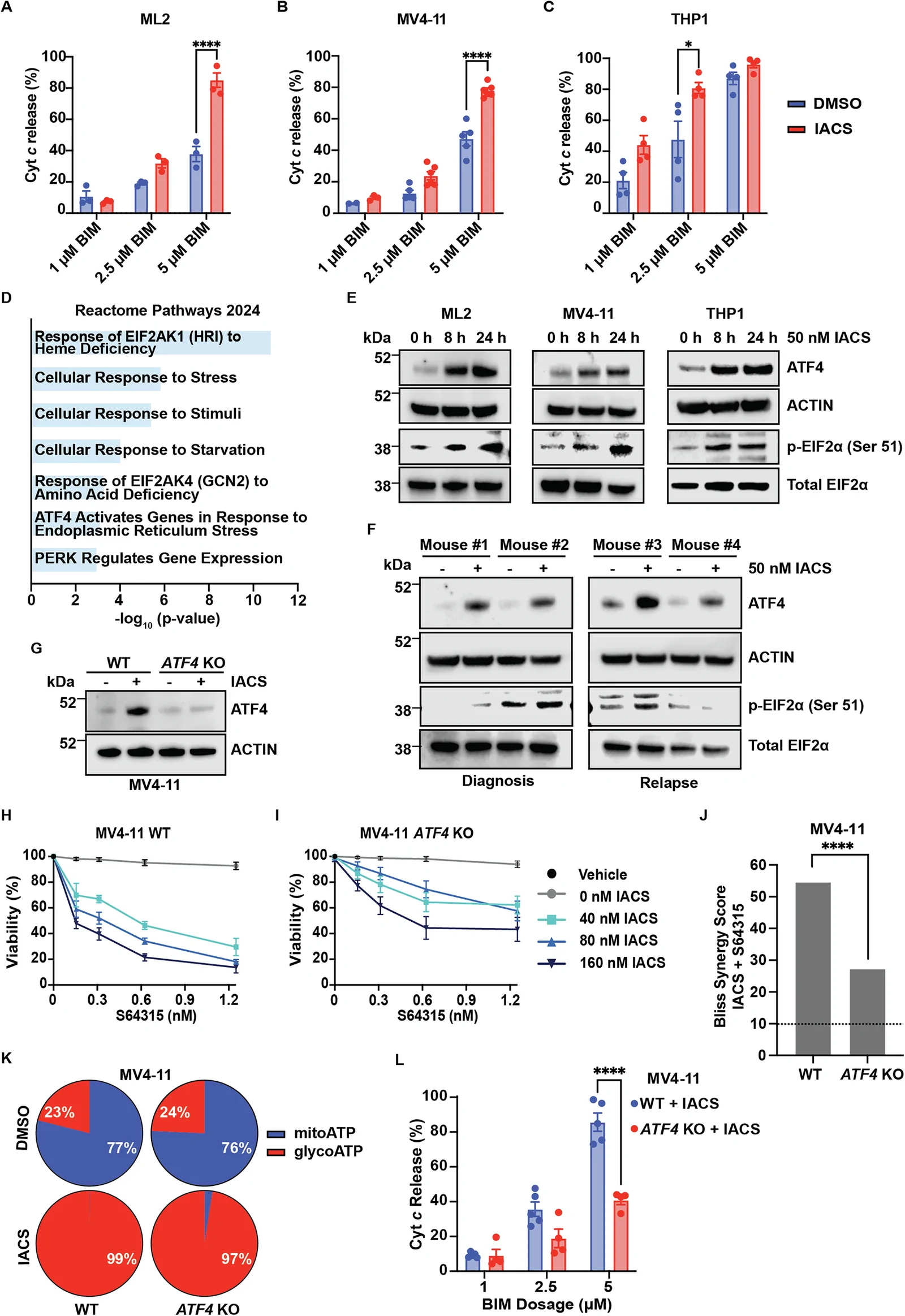
IACS-010759 + MCL-1i induces cell death through ATF4 activation. Dynamic BH3 profiling of ML2 (**A**), MV4-11 (**B**), and THP1 (**C**) cells treated with 50 nM IACS-010759 (IACS) for 24 h. Statistical analysis performed using two-way ANOVA of three or more independent experiments. Bars show the mean, with individual data points plotted for each sample. Error bars represent the SEM. *P*-values < 0.05 are indicated by * and *p*-values ≤ 0.0001 are indicated by ****. **D** Enrichr analysis of the top 30 upregulated indexed hits identified by RNA sequencing after IACS treatment in MV4-11 and THP1 cells. The indexed hits rank the top 30 enriched transcripts amongst both cell lines and treatment times with a *p*-value < 0.05 and |log2R| > 0.585 (half-fold change). **E** Western blot analysis of ATF4 activation and p-EIF2α in acute myeloid leukemia cell lines treated with 50 nM IACS for 8 or 24 h. **F** Western blot analysis of ATF4 activation and p-EIF2α in PDX cells treated with 50 nM IACS for 24 h ex vivo. **G** Western blot of WT and *ATF4* KO MV4-11 cell lines treated with IACS for 24 h to validate loss of ATF4 expression. Cell viability graphs (AV^−^/PI^−^) of wild-type (WT, **H**) and *ATF4* knockout (KO, **I**) MV4-11 cells treated with increasing doses of IACS and S64315. Cell viability is normalized to the vehicle-treated (DMSO) control, defined as 100%. Graphs represent data from three or more independent experiments, and error bars represent the SEM. **J** Bliss synergy scores of WT and *ATF4* KO MV4-11 cells. The dashed line indicates values < 10 as synergistic. Statistical analysis shows *Zp*-value comparing Bliss scores and *p*-values between the WT and *ATF4* KO MV4-11 cells. *Zp*-values ≤ 0.0001 are indicated by ****. **K** Calculated ATP production graphs of WT and *ATF4* KO MV4-11 cells treated with DMSO or IACS for 1 h before undergoing the Seahorse Mito Stress Test to determine the estimated ATP production from OXPHOS (mitoATP) and glycolysis (glycoATP). Values represent calculated estimates of ATP production, not direct ATP measurements. **L** dBH3 profiling of WT or *ATF4* KO MV4-11 cells treated with DMSO or 50 nM IACS. Bars show the mean, with individual data points plotted for each sample. Error bars represent the SEM. Two-way ANOVA performed on three or more dBH3 profiling experiments. *P*-values ≤ 0.0001 are indicated by ****.

Mitochondrial priming describes how near a cell is to reaching the apoptotic threshold; specifically, how “ready” its mitochondria are to undergo MOMP in response to a pro-apoptotic signal [76]. To better understand the mechanism of how IACS potentiates the effects of MCL-1 inhibitors, we tested the effects of IACS on mitochondrial priming using dynamic BH3 (dBH3) profiling, which tests drug-induced MOMP through the observation of Cyt *c* release [77]. Three *KMT2A*-r AML cell lines were treated with IACS for 24 h, permeabilized with digitonin, and then exposed to a BIM peptide that mimics BIM’s BH3 domain, which binds to all the anti-apoptotic BCL-2 family members and causes Cyt *c* release [78]. Titration of the BIM peptide up to 5 µM revealed that Cyt *c* was more readily released from the mitochondria after IACS treatment when compared to cells treated with DMSO (Fig. 3A–C). The readiness of IACS-treated samples to release Cyt *c* indicates that the cells were more primed after culture in IACS, making them more susceptible to apoptosis-inducing stimuli.

### IACS induces the ISR to activate ATF4

To mechanistically interrogate how IACS primes *KMT2A*-r AML cells to the effects of MCL-1 inhibition, THP1 and MV4-11 cell lines were treated with 50 nM IACS for either 8 h or 24 h and RNA sequencing was performed. Enrichr analysis of the top 30 indexed hits (across both cell lines and timepoints) revealed that IACS treatment elicited gene expression patterns associated with the ISR, including those associated with *EIF2AK1* (protein produce referred to as HRI), *EIF2AK4* (protein produce referred to as GCN2), and *EIF2AK3* (protein produce referred to as PERK) kinase activated pathways (Fig. 3D). Furthermore, Enrichr pathway analysis of the most significant hits at 8 h and 24 h following IACS treatment in both cell lines revealed enrichment of HRI-mediated ISR signaling and glucose metabolism pathways (Fig. S9A–D). The upregulation of glucose-associated pathways in the MV4-11 cells following 24 h of IACS treatment is consistent with the increased reliance on glycolysis quantified and calculated using Seahorse Mito Stress Tests across all cell lines (Fig. 2B). Taken together, the RNA sequencing indicates that IACS activates the ISR as a single agent.

To verify the activation of the ISR after IACS treatment, the *KMT2A*-r AML cell lines were treated with 50 nM IACS for 8 h or 24 h and Western blotted to assess canonical ISR activation by phosphorylation of EIF2α at serine 51 (p-EIF2α) and ATF4 induction. Both p-EIF2α and ATF4 expression were increased in all three AML cell lines after IACS treatment (Fig. 3E). While ATF4 was also induced in all the *KMT2A*-r PDX cells treated with IACS ex vivo for 24 h, p-EIF2α was only detected in three of the four samples (Fig. 3F). These data indicate that IACS treatment activates the ISR.

To investigate if activation of ATF4 by the co-treatment of IACS and MCL-1 inhibitors had a role in inducing apoptosis, an *ATF4* KO MV4-11 cell pool was generated via CRISPR gene editing, and loss of protein expression was validated after IACS treatment (Fig. 3G). After 24 h of co-treatment with IACS and S64315, the *ATF4* KO MV4-11 cells were less responsive to the combination treatment of IACS and S64315 than WT MV4-11 cells (Fig. 3H, I); the Bliss synergy score associated with the co-treatment of *ATF4* KO MV4-11 cells was also significantly decreased (Fig. 3J). To ensure the loss of ATF4 still rendered the cells responsive to IACS treatment, MV4-11 WT and *ATF4* KO cells were treated with 50 nM IACS for 1 h, and cellular respiration was assessed. Even in the absence of ATF4, IACS treatment decreased the calculated mitoATP production (Figs. 3K and S10), indicating that IACS treatment inhibits CI independently of ATF4. Since the *ATF4* KO MV4-11 cells were more resistant to the combined effects of IACS and S64315, we wanted to test whether IACS treatment resulted in differential mitochondrial priming in the *ATF4* KO cells. After IACS exposure, the absence of ATF4 decreased the Cyt *c* release when compared to WT (Fig. 3L), indicating a reduction in overall mitochondrial priming. These results indicate that ATF4 induction triggered by IACS treatment promotes apoptotic priming to MCL-1 inhibitors in *KMT2A*-r AML cells.

### IACS activates ATF4 primarily through the DELE1-HRI pathway

**Fig. 4:**
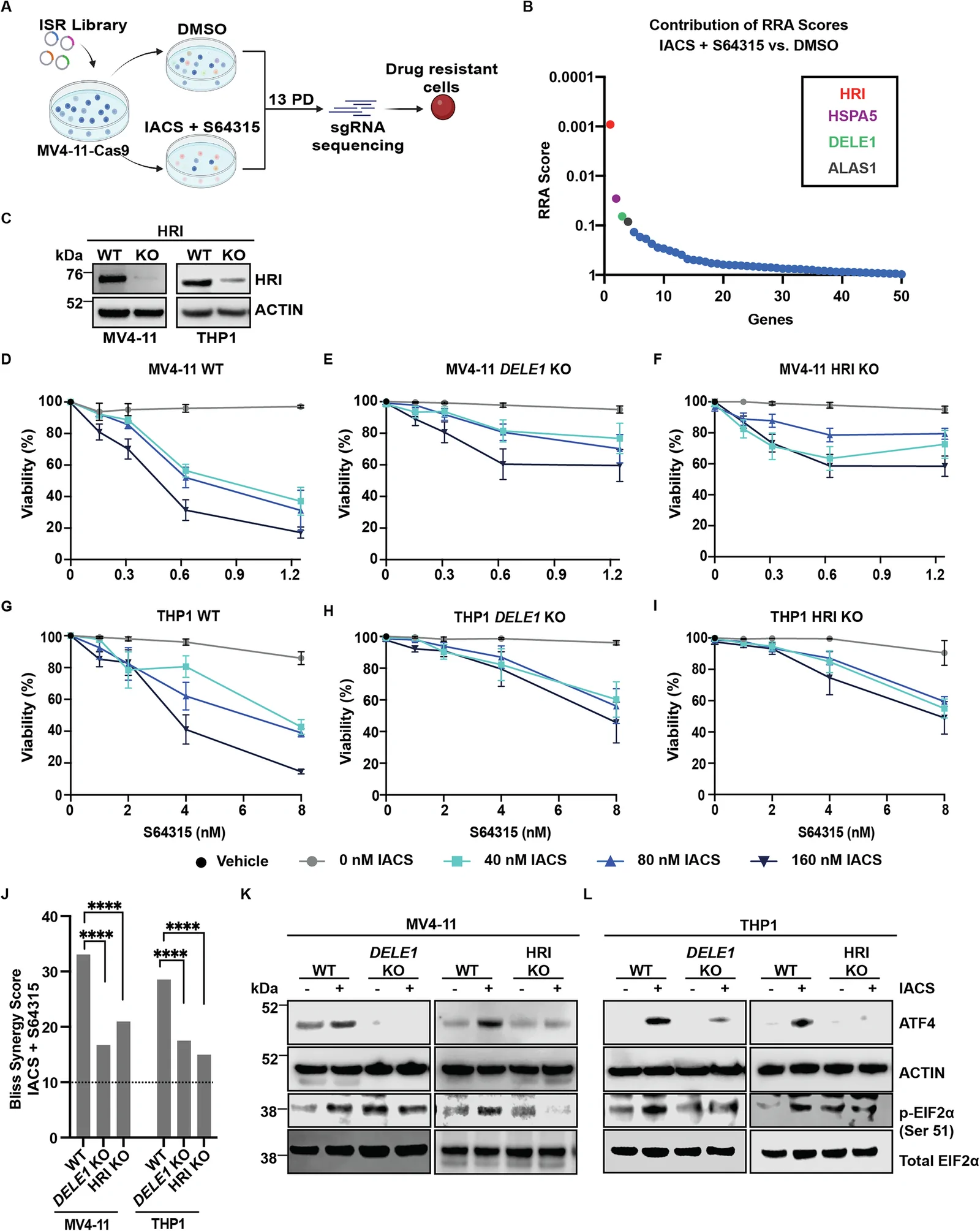
CRISPR Knockout (KO) screen focused on integrated stress response indicates loss of DELE1-HRI axis promotes combination therapy resistance. **A** Schematic of screen showing Cas9 expressing MV4-11 cells transduced with the integrated stress response CRISPR knockout (KO) library were treated with either DMSO or co-treated with 4 nM S64315 and 160 nM IACS-010759 (IACS). Drugs were washed from the cells and further cultured for 13 population doublings (PD) and sent for Illumina sequencing and analyzed using MAGeCK-VISPR to identify genes that lead to combination treatment resistance. **B** Contribution of robust rank aggregation scores of the top hits from the analysis of the combined treatment of S64315 and IACS vs. DMSO-treated samples. **C** Expression levels of HRI in MV4-11 and THP1 KO independent cell lines. Cell viability (AV^-^/PI^-^) graphs of WT (**D** and **G**), *DELE1* KO (**E** and **H**), and HRI KO (**F** and **I**) MV4-11 (**D**–**F**) and THP1 (**G**–**I**) cell lines treated with IACS and S64315. Graphs represent data from three more independent experiments, and error bars represent the SEM. Cell viability is normalized to a DMSO control, defined as 100%. **J** Bliss synergy scores corresponding to the graphs in **D**–**I**. The dashed line indicates values over 10 as synergistic. Statistical analysis shows *Zp*-values comparing Bliss synergy scores and *p*-values between the WT and KOs. *Zp*-values ≤ 0.0001 are indicated by ****. ATF4 and p-EIF2α expression after IACS treatment in *DELE1* or HRI KO MV4-11 (**K**) and THP1 (**L**) cells. Independent KO cell lines presented in this figure are the primary cell lines and labeled as L1 in Supplementary (S) figures.

To determine the mechanism by which IACS stimulates mitochondrial priming, we sought to identify genes that, when lost, conferred resistance to the co-treatment of IACS and MCL-1 inhibitors. An ISR-focused CRISPR KO library was generated containing guides targeting the four eIF2α kinases, pro-apoptotic genes, and the top 30 upregulated and 5 downregulated transcripts identified by the RNA sequencing, which included 25 predicted or validated ATF4 target genes (Table S1). The ISR KO library was transduced into MV4-11 cells and treated with DMSO or a combination of 160 nM IACS and 4 nM S64315, resulting in ~70% cell death (Fig. 4A). The drugs were washed out after 24 h, and the cells were cultured in drug-free media for 13 population doublings. RRA by MAGeCK-VISPR analysis revealed that the loss of the eIF2α kinase, HRI, *DELE1* (HRI activator), *HSPA5* (ER chaperone), and *ALAS1* (heme biosynthesis enzyme) promoted drug resistance to IACS and S64315 (Fig. 4B). Together, the ISR CRISPR screen implicated the DELE-HRI axis of the ISR as key pro-death signaling pathway when IACS and MCL-1 inhibitors are combined.

DELE1 relays mitochondrial stress to the cytosol by activating the kinase HRI, which initiates ISR signaling [38, 39]. Based on the results from our ISR CRISPR screen, we hypothesized that the DELE1-HRI pathway was responsible for the pro-apoptotic signaling when IACS is combined with MCL-1 inhibitors. To test this possibility, we knocked out *DELE1* or HRI in the MV4-11 and THP1 cell lines using CRISPR gene editing and examined 2-3 independent lines (L) per genotype. There are no good antibodies to detect endogenous DELE1 and a minor amount of residual HRI protein was detected in the lines by Western blot (Fig. 4C). To functionally assess whether the DELE1-HRI pathway was abolished, *KMT2A*-r AML cell lines lacking *DELE1* or HRI were treated with the mitochondrial uncoupler FCCP to activate the DELE1-HRI-ATF4 signaling axis. The *KMT2A*-r AML cell lines lacking *DELE1* or HRI failed to induce ATF4 expression (Fig. S11A, B), indicating an impaired DELE1-HRI signaling axis in the CRISPR-edited lines. IACS pretreatment for 1 h in either *DELE1* or HRI KO cells resulted in the rapid repression of the calculated mitoATP production by Seahorse Mito Stress Test (Figs. S12 and S13). Like the response observed in the *ATF4* KO cells, these data support the ability of IACS to repress CI, independently of the activation of the ISR.

Furthermore, the loss of *DELE1* (Figs. 4E, H and S14A, D, E) or HRI (Figs. 4F, I and S14B, F, G) substantially blunted the cell death induced in *KMT2A*-r AML cell lines by the combination of IACS and S64315 when compared to the WT cell lines (Fig. 4D, G). *Zp*-vales calculated from the Bliss synergy scores in both MV4-11 and THP1 cells indicated the loss of *DELE1* or HRI (Figs. 4J and S14C, H) caused a significant reduction in the Bliss synergy scores compared to Bliss synergy scored in the WT MV4-11 and THP1 cells (Fig. 4J). Furthermore, the absence of *DELE1* or HRI blunted the ATF4 activation in response to IACS (Figs. 4K, L and S14I, J). Despite the observed repression of ATF4 activation, p-EIF2α expression was observed in the KOs (Figs. 4K, L and S14I, J), indicating their absence alone can induce its phosphorylation, consistent with a prior report [38]. Together, these data suggest the DELE1-HRI-ATF4 signaling pathway induces a pro-apoptotic response when IACS is combined with S64315.

### Co-treatment with IACS and S64315 induces an anti-leukemic response in vivo

**Fig. 5:**
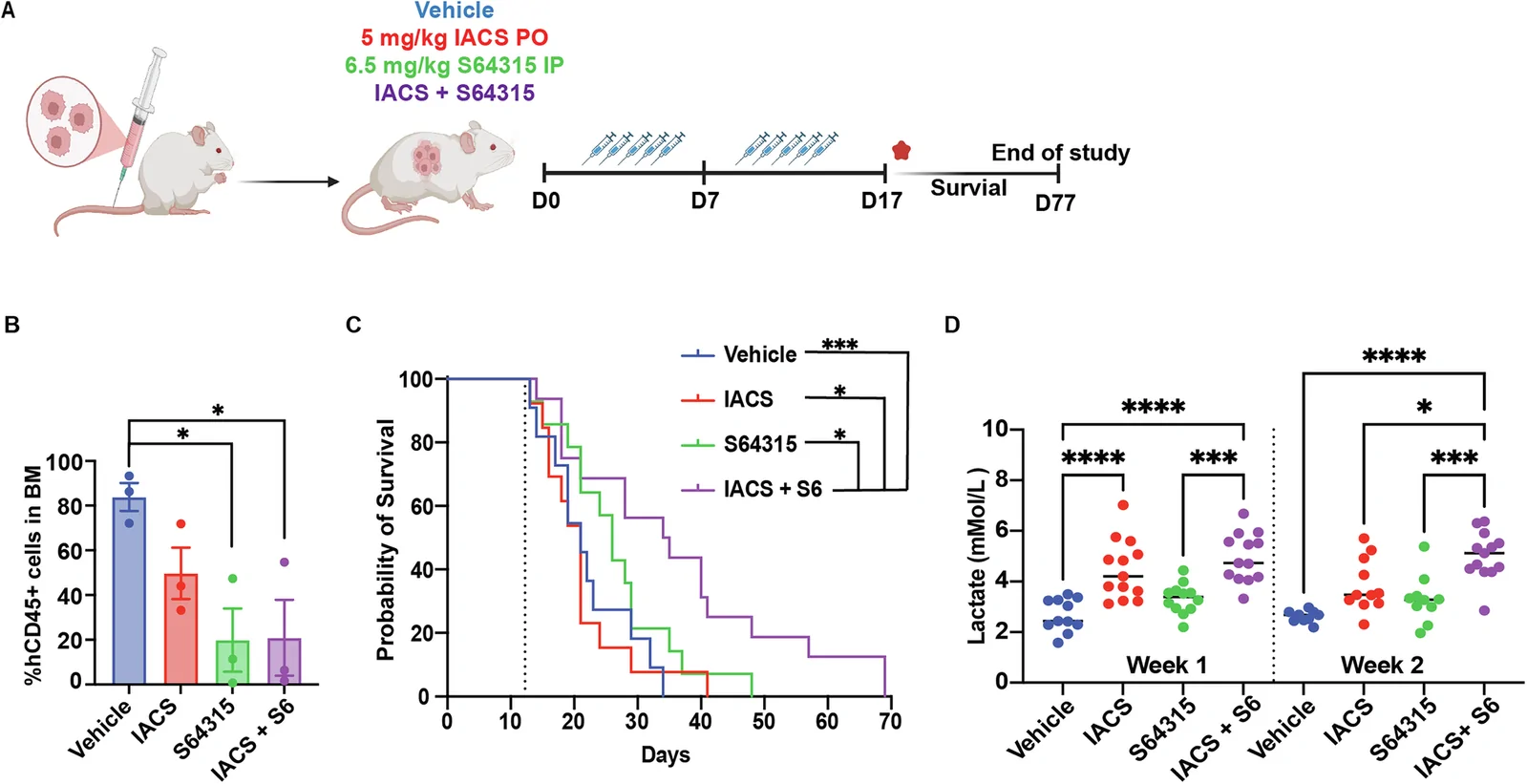
IACS-010759 and S64315 induce an anti-leukemic effect in vivo*.* **A** Schematic of in vivo experiments. Patient-derived xenograft cells were injected into NSG-SGM3 mice by tail vein injection. After engraftment, mice were treated with the indicated doses of IACS-010759 (IACS), S64315, or combination for 5 days on and 2 days off for two cycles (denoted by the syringes). After the end of the cycles, a partial take-down (denoted by the red star) was performed of three animals and the remaining animals were kept on the study for survival analyses. **B** hCD45^+^ cells in the bone marrow at the end of treatment. Bars represent the mean, with individual data points shown for each sample (*n* = 3 mice/group). Error bars represent the SEM. Statistical significance was determined by one-way ANOVA. *P*-values ${\boldsymbol{\le }}$ 0.05 are indicated by *. **C** Survival analysis of mice treated with vehicle, IACS, S64315, or IACS combined with S64315. Statistical significance of the Kaplan–Meier Curve was calculated using the Fleming–Harrington test for right-censored data. The dashed line indicates the last treatment administered (day 12). **D** Lactate readings from blood plasma samples after one or two weeks on treatment. The scatter dot plot positions the line at the sample median with individual datapoints shown. Statistical significance was determined using a one-way ANOVA. *P*-values ${\boldsymbol{\le }}$ 0.05 are indicated by *, ${\boldsymbol{\le }}$ 0.001 are indicated by ***, and ${\boldsymbol{\le }}$0.0001 are indicated by ****.

To explore the translational potential of combining MCL-1 inhibitors with IACS in vivo, *KMT2A-MLLT3* AML PDX cells, derived from a patient with relapsed disease, were expanded in NSG-SGM3 mice. Once the mice showed approximately 0.5% hCD45^+^ cells in the peripheral blood, they were randomized for treatment with vehicles, IACS (5 mg/kg by oral gavage and vehicle for S64315, once daily), S64315 (6.5 mg/kg by intraperitoneal injection and vehicle for IACS, once daily), or by combination treatment for a 5-day on/2-day off regimen for 2 weeks (Fig. 5A). At the end of the second treatment cycle, 3 mice per group were euthanized for analysis. From this cohort, flow cytometric assessment of the bone marrow showed a significant decrease in the hCD45^+^ cells present in the bone marrow in both the S64315 monotherapy and co-treatment groups (Fig. 5B).

After the two treatment cycles, the remaining mice were monitored twice daily for up to 2 months for survival studies. Once the mice reached a humane endpoint (i.e., hind leg paresis, tachypneic, delayed response, hunched posture, and weight loss), they were euthanized, and the survival post-treatment was recorded and analyzed. The lifespan of the mice that received the combination therapy was extended significantly with a median survival of 34.5 days (Fig. 5C). The vehicle and IACS cohorts had a median survival of 21 days, indicating a lack of efficacy for IACS as a single agent. Mice that received S64315 treatment had a minor survival advantage over the vehicle- and IACS-treated cohorts, with median survivals of 26 days. Despite the extended survival of the combination-treated mice, all mice eventually exhibited physical symptoms of leukemia and reached the defined humane endpoint.

IACS treatment has been reported to induce toxicities during clinical trials, including lactic acidosis and peripheral neuropathies [26]. To determine whether our treatment with IACS and/or MCL-1 inhibitors caused such toxicities in vivo, we assessed blood serum lactate levels in at least nine mice per group after 1 or 2 weeks on treatment (Fig. 5D). The S64315-treated cohort had similar levels of lactate when compared to DMSO. Elevated lactate levels were observed in both the IACS- and combination-treated cohorts, indicating IACS repressed OXPHOS in vivo. Mice treated with IACS as either a monotherapy or combination did not reach the 10 mM serum lactate levels seen in metformin-induced models of lactic acidosis [79]. However, mice treated with 5 mg/kg IACS exhibited serum lactate levels comparable to those previously reported, in which peripheral neuropathy was observed [26].

## Discussion

MCL-1 inhibiting BH3 mimetics constitute a compelling class of anticancer agents because MCL-1 antagonizes apoptosis and drives both tumor maintenance and resistance to conventional therapies [10, 80–83]. However, the clinical development of MCL-1 inhibitors has been hindered by dose-limiting toxicities, such as cardiac damage, that restrict effective drug exposure in phase I trials [84]. To maximize therapeutic benefit while minimizing toxicity, we sought to identify combination strategies that enhance the efficacy of MCL-1 inhibition at lower doses. To identify strategies that potentiate the effects of MCL-1 inhibition, a metabolic CRISPR knockout screen revealed that loss of CI components or assembly factors sensitized AML cells to MCL-1 inhibition. As a proof-of-concept, pharmacologic CI inhibition with IACS synergized with multiple MCL-1 inhibitors to trigger robust apoptosis in *KMT2A-*r AML cell lines and patient-derived xenografts. Mechanistically, IACS increased mitochondrial apoptotic priming and activated the ISR via ATF4, whose loss diminished combination-induced apoptosis. CRISPR screening and genetic validation identified the DELE1-HRI axis as the upstream mediator of IACS-induced ATF4 activation and potentiated cell death. Finally, co-treatment with IACS and an MCL-1 inhibitor extended survival and reduced leukemic burden in *KMT2A-*r AML xenograft models, establishing mitochondrial CI inhibition as a metabolic vulnerability that enhances MCL-1-targeted therapy.

Aside from the hits in the ETC, our metabolic CRISPR KO screen implicated loss of aspects of lipid metabolism as potential sensitizers of AML cells to MCL-1 inhibition (Fig. 1B, C). Both our group and others have identified roles for MCL-1 in promoting fatty acid β-oxidation, highlighting the potential for inhibiting this function to promote responses to MCL-1 inhibition [70, 71]. Furthermore, either genetic loss or pharmacologic inhibition of MCL-1 has been shown to impact OXPHOS [74, 85]. This posits the question of whether the synergy observed by combining MCL-1 inhibitors with IACS reflects inhibition of independent pathways or convergent effects on mitochondrial metabolism. Our data demonstrate that IACS potently inhibits ETC function, but fails to induce cell death as a single agent. Thus, almost complete repression of ETC function through CI inhibition is insufficient to induce AML killing in the time frames tested. In contrast, while MCL-1 inhibitors can repress mitochondrial metabolism and directly promote MOMP, MCL-1 inhibition alone at the doses tested was insufficient to induce AML death. Therefore, the repression of ETC induced by IACS appears to increase the priming of the AML cells, making them respond more robustly to MCL-1 inhibition. While there may be overlap between MCL-1 inhibition and IACS in repressing OXPHOS, the synergism of the combination treatment seems to reflect that IACS potently represses ETC function, increasing priming of AML cells to MCL-1 inhibition.

Our findings further demonstrate that the increased apoptotic priming induced by CI inhibition depends on the stress-responsive transcription factor ATF4. Notably, ATF4 plays a dual role in leukemogenesis, acting both as a mediator of leukemic adaptation and as a potential therapeutic effector. Under basal or oncogenic stress conditions, ATF4 supports leukemic cell survival by maintaining amino acid metabolism, redox balance, and mitochondrial homeostasis, thereby promoting leukemogenesis, disease persistence, and therapeutic resistance [44]. In contrast, when excessively or acutely activated by mitochondrial or metabolic stress, ATF4 drives pro-apoptotic signaling that enhances therapeutic responses, particularly in contexts where the ISR is pharmacologically engaged to overcome leukemic resistance [56, 86, 87]. Although ATF4 is known to regulate diverse adaptive responses to metabolic and proteotoxic stress, the downstream mechanisms by which it modulates mitochondrial priming in response to CI inhibition remain unclear. Our RNA sequencing data did not reveal major changes in the transcriptome of the BCL-2 family proteins induced by IACS treatment. Given that post-translational modifications of BCL-2 family proteins can fine-tune the balance between pro- and anti-apoptotic interactions [88–90], such modifications may promote alterations in apoptotic priming [91]. Alternatively, an as-yet-unidentified regulator of apoptotic priming may contribute to the modulation of mitochondrial sensitivity following CI inhibition. Together, these findings suggest that ATF4-dependent reprogramming of mitochondrial priming after IACS treatment may involve mechanisms beyond changes in BCL-2 family expression. Further studies are needed to clarify how metabolic stress influences apoptotic susceptibility.

ATF4 can be induced through phosphorylation of eIF2α by any of four stress-responsive kinases, PERK (ER stress), GCN2 (amino acid deprivation), HRI (mitochondrial or heme stress), or PKR (viral infection or double-stranded RNA), each activating the ISR under distinct cellular conditions [92–98]. Mechanistically, we identify that repression of CI function activates the DELE1-HRI mitochondrial stress pathway that induces ATF4. DELE1, initially identified as a DAP3 binding molecule that promotes apoptosis and later as an activator of HRI-mediated stress signaling, has emerged as a central mediator of the mitochondrial stress response in both normal and malignant contexts [38, 39, 99]. In normal tissues, DELE1 primarily supports protective adaptation; studies in skeletal and heart muscle demonstrated that while DELE1 is dispensable under basal conditions, its loss exacerbates pathology in models of mitochondrial myopathy [100, 101]. In cancer, however, DELE1 appears to have context-dependent roles: it is elevated in colorectal cancer and linked to resistance to fluorouracil and oxaliplatin [102]. However, *DELE1* is frequently deleted in -5/del(5q) AML, where its loss confers resistance to mitochondrial stressors [103]. Our findings indicate that combining IACS with MCL-1 inhibition engages mitochondrial stress through DELE1-HRI signaling, paralleling the resistance observed in *DELE1*-deficient 5/del(5q) leukemia and highlighting DELE1’s capacity to facilitate apoptosis under mitochondrial stress. These results suggest that exploiting DELE1-mediated ATF4 activation could enhance cancer cell death and potentially improve responses to both traditional and targeted therapies.

While the loss of *DELE1* and HRI promotes resistance to the combination of IACS and S64315, our results do not show a complete attenuation of cell death in their absence. The ISR consists of three kinases besides HRI: PKR, PERK and GCN2, all of which can phosphorylate eIF2α under stressful conditions. Though the loss of DELE1 or HRI prevents or reduces ATF4 activation in response to IACS, it cannot be ruled out that another ISR kinase is responsible for ATF4 activation. While the studies presented here focus on the role of HRI in cell death induced by the combination of IACS and S64315, a stringent analysis of other kinases should be performed to determine if any compensatory effects by the other ISR kinases are involved. Furthermore, the loss of ATF4 also promoted more resistance to cell death induced by the combination treatment than either the loss of DELE1 or HRI alone. Ultimately, the only genetic KO that completely attenuated the observed cell death was the absence of both BAK and BAX. Taken together, these data indicate that apoptosis is occurring, but may be only partially modulated by activation of the ISR. Additional studies are needed to define the pathway(s) that act in parallel with the ISR to promote cell death in response to combined treatment with IACS and MCL-1 inhibition.

The mitochondrial stressors FCCP and oligomycin contributed to the discovery of the ISR’s OMA1-DELE1-HRI arm [38, 39]. However, inhibition of ETC complexes can also activate ATF4 through the mitochondrial ISR. While we used IACS to induce ATF4 activation, other CI inhibitors, such as metformin and rotenone, have also been shown to activate ATF4 [87, 104, 105]. Furthermore, inhibition of complex II and complex III similarly triggers ATF4 activation [106–108]. Although less extensively studied, perturbation of complex IV activity, by agents such as carbon monoxide or arsenic trioxide, can also induce ATF4. Several ETC inhibitors, particularly those targeting CI (metformin, phenformin, IACS, IM156) or complex III (atovaquone), have been tested as cancer therapies, either alone or in combination with standard chemotherapy [33–37, 109, 110]. Direct inhibition of complex IV remains challenging due to toxicity, though agents that indirectly disrupt mitochondrial function, such as arsenic trioxide, are FDA-approved for certain leukemias. Although we did not explore this question here, we hypothesize that any ETC impairment sufficient to activate ATF4 would synergize with BH3 mimetics. This opens the possibility of multiple potential methods to repress ETC function to improve responses in poor-prognosis AML.

IACS and MCL-1-targeting BH3 mimetics have shown antitumor efficacy in preclinical models against hematologic malignancies; however, their translation to clinical use has been hindered by dose-limiting toxicities. IACS is associated with dose-limiting hematologic adverse events, including neutropenia, thrombocytopenia, and anemia, and has been linked to neurological complications, such as peripheral neuropathy and cognitive impairment, often attributed to lactic acidosis [26]. Consistent with prior studies, administration of IACS, either as a single agent or in combination with MCL-1 inhibition, resulted in elevated serum lactate levels in treated mice [26]; however, these levels did not reach thresholds defined for lactic acidosis in murine models, and no overt peripheral neuropathy was observed [111, 112]. Our findings further indicate that brief, low-dose exposure to IACS in combination with S64315 was sufficient to extend survival in mice with *KMT2A-*r AML, suggesting that prolonged high-level IACS exposure may not be required for efficacy. Importantly, this short-course regimen mitigated toxicities historically associated with MCL-1 inhibitors and IACS, including myelosuppression, lactic acidosis, and peripheral neuropathies [26]. A limitation of this study is that extended dosing schedules were not evaluated, which may further enhance combinatorial efficacy but may also increase the risk of adverse events. Moreover, the higher affinity of MCL-1 inhibitors for human compared to murine MCL-1 complicates toxicity assessment in mouse models [13, 113].

In summary, our results highlight a therapeutic strategy to enhance the efficacy of MCL-1 inhibitors in AML by co-administering IACS to activate a pro-apoptotic ISR. By leveraging mitochondrial stress-induced apoptotic signaling, this combination approach has the potential to overcome resistance mechanisms and selectively sensitize AML cells to BH3 mimetic therapy. Importantly, this strategy may provide a means to improve treatment outcomes in high-risk or refractory AML, a patient population with limited therapeutic options. Future studies are warranted to validate these findings in additional in vivo models, define the underlying molecular mechanisms in greater detail, and assess the safety and translational potential of this combination for clinical application. Such investigations will be critical in establishing the feasibility of targeting stress-induced apoptosis as a complementary approach to existing AML therapies.

## Supplementary information

Sup Table 1

Sup Table 2

Sup Table 3

Sup Fig 1

Sup Fig 2

Sup Fig 3

Sup Fig 4

Sup Fig 5

Sup Fig 6

Sup Fig 7

Sup Fig 8

Sup Fig 9

Sup Fig 10

Sup Fig 11

Sup Fig 12

Sup Fig 13

Sup Fig 14

Sup Fig 15 UNCROPPED WESTERNS

Sup Figures legend

## Data Availability

CRISPR KO screen raw sequencing data is archived in NIH BioProject under Accession SUB15754309 and will be made publicly available upon publication. The RNA sequencing data has been deposited in NCBI’s Gene Expression Omnibus, and is accessible through GEO Series accession number (GSE309897), and will be made publicly available upon publication.
