# Supplementary material for: Mitochondrial integrated stress response activation creates a therapeutic vulnerability to MCL-1 inhibition in acute myeloid leukemia
Source: Cell Death Dis. 2026 Jun 25;17(1):785. doi: 10.1038/s41419-026-09037-w (PMC13558565; doi:10.1038/s41419-026-09037-w)
Supplement: Supplementary file 1 [file 41419_2026_9037_MOESM18_ESM.pdf]

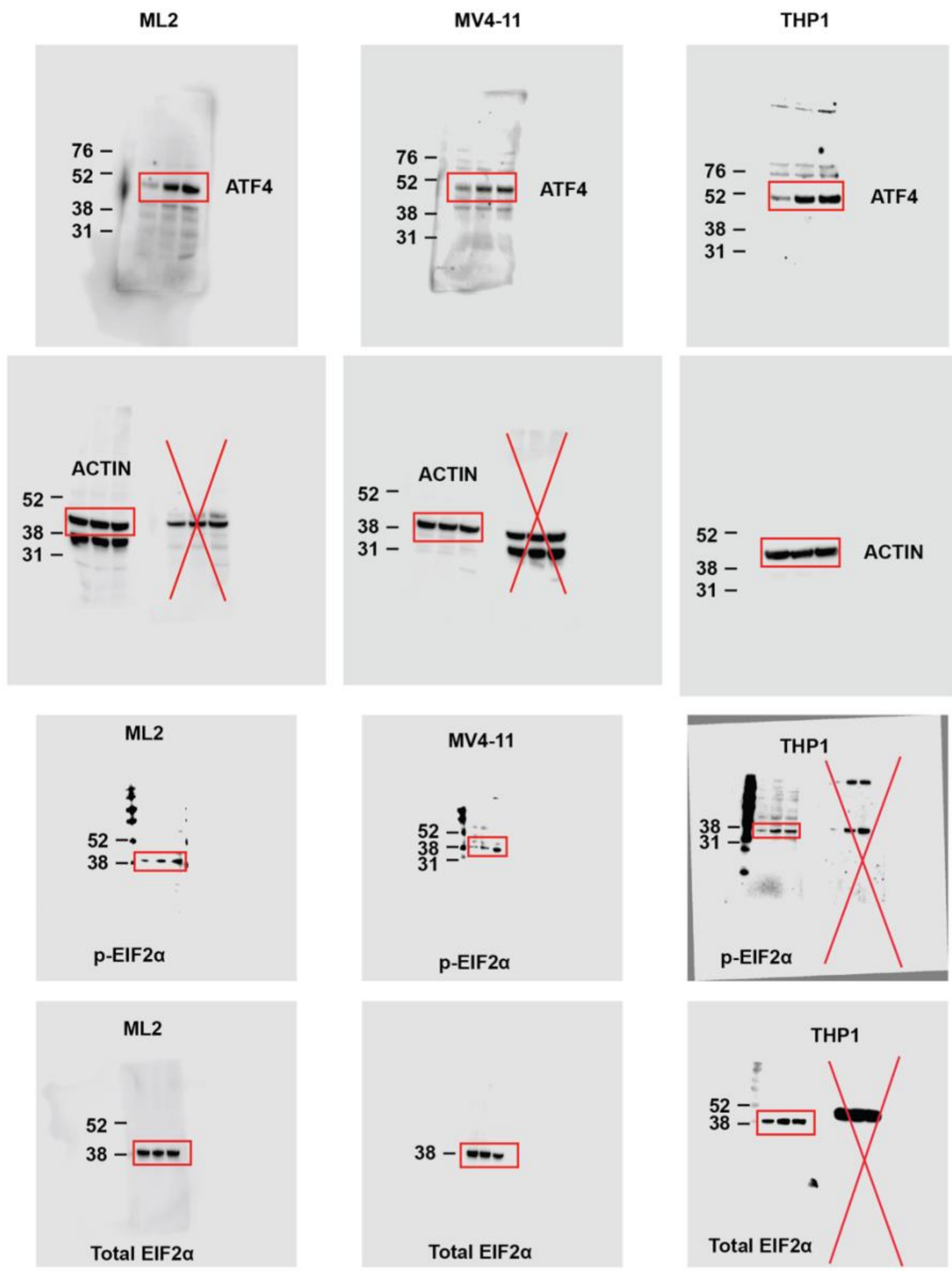

Supplementary Fig. S15A: Uncropped western blots for Fig. 3E.

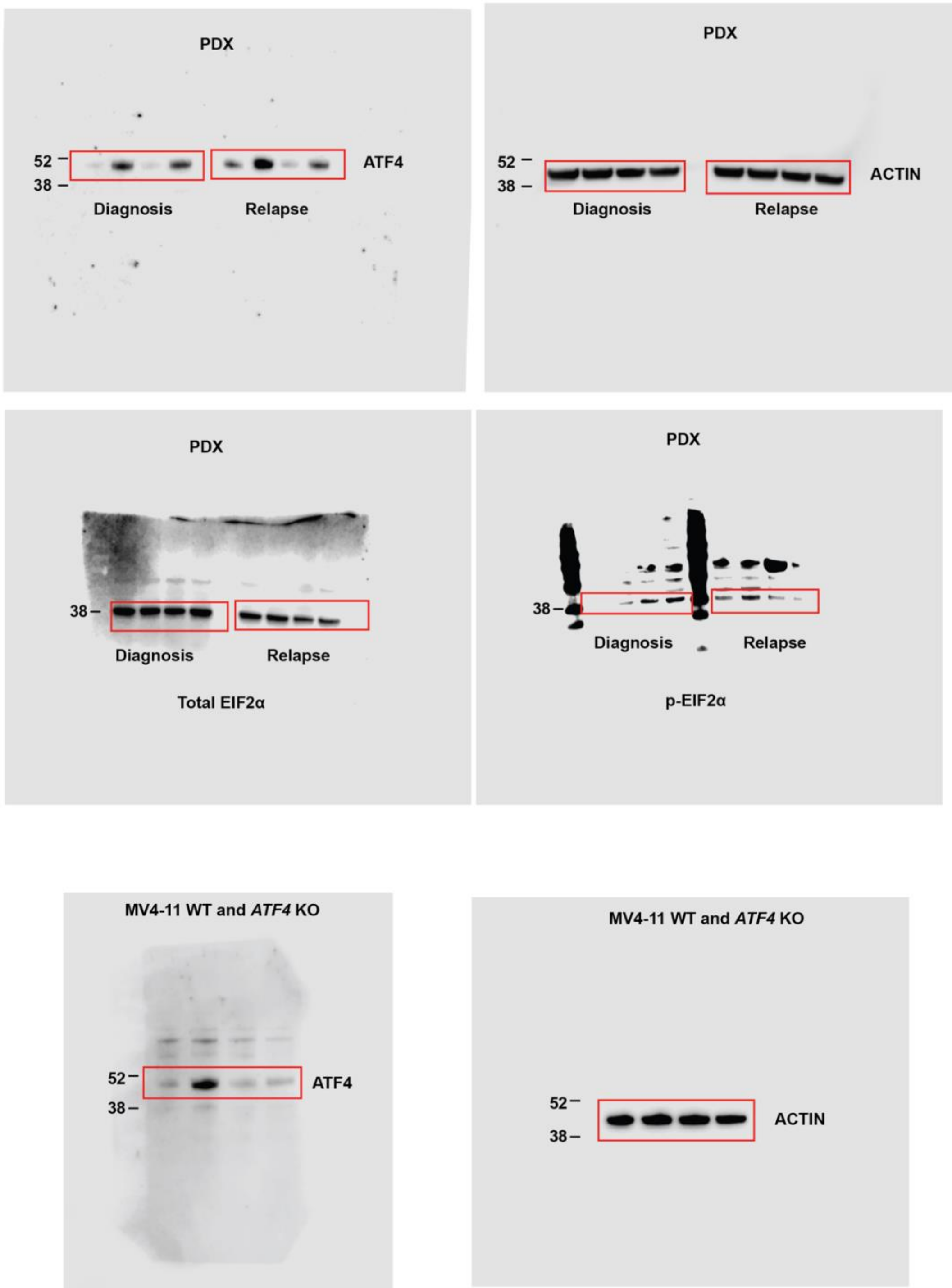

Supplementary Fig. S15B: Uncropped western blots for Fig 3F and G.

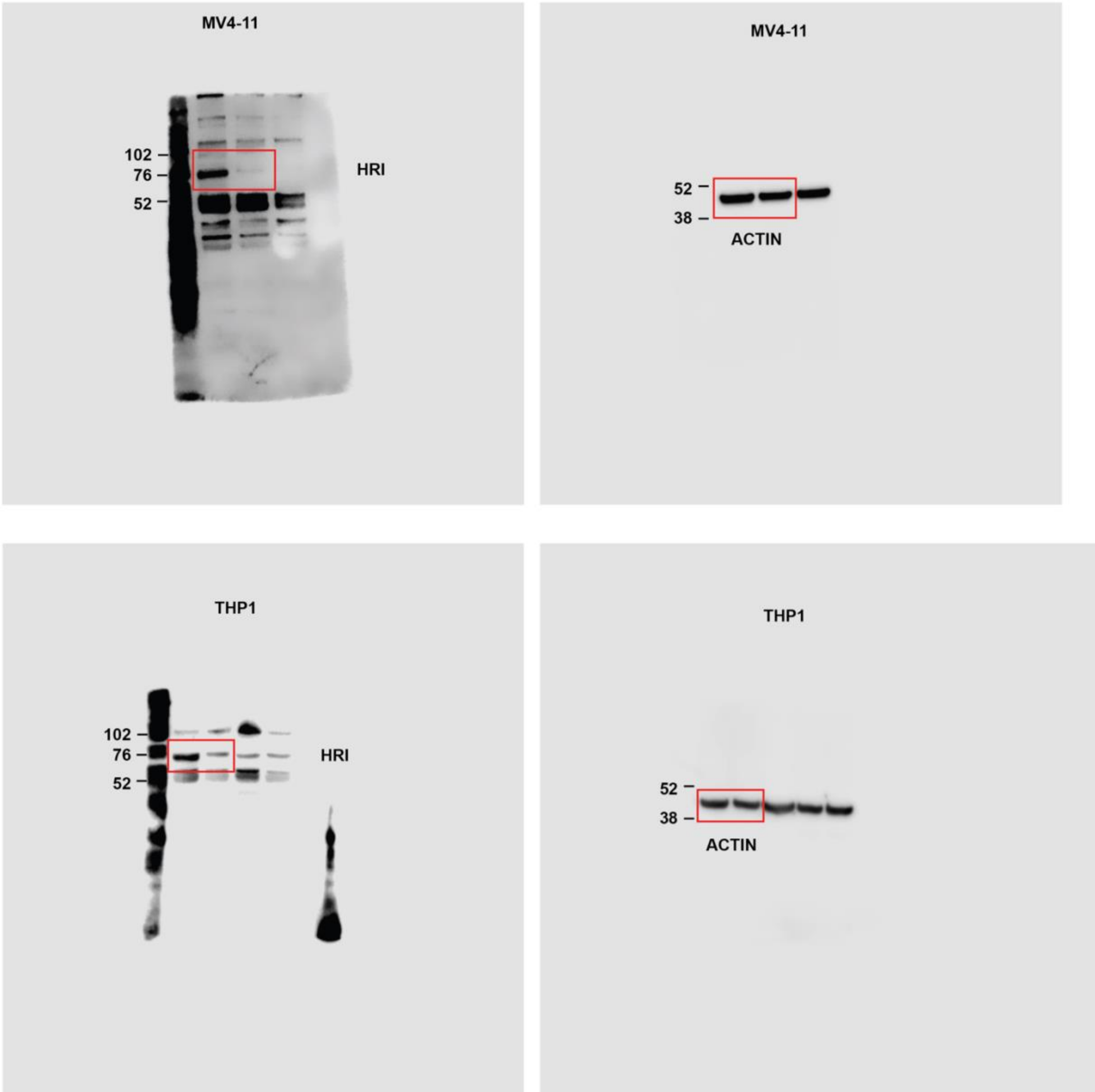

Supplementary Fig. S15C: Uncropped western blots for figure 4C.

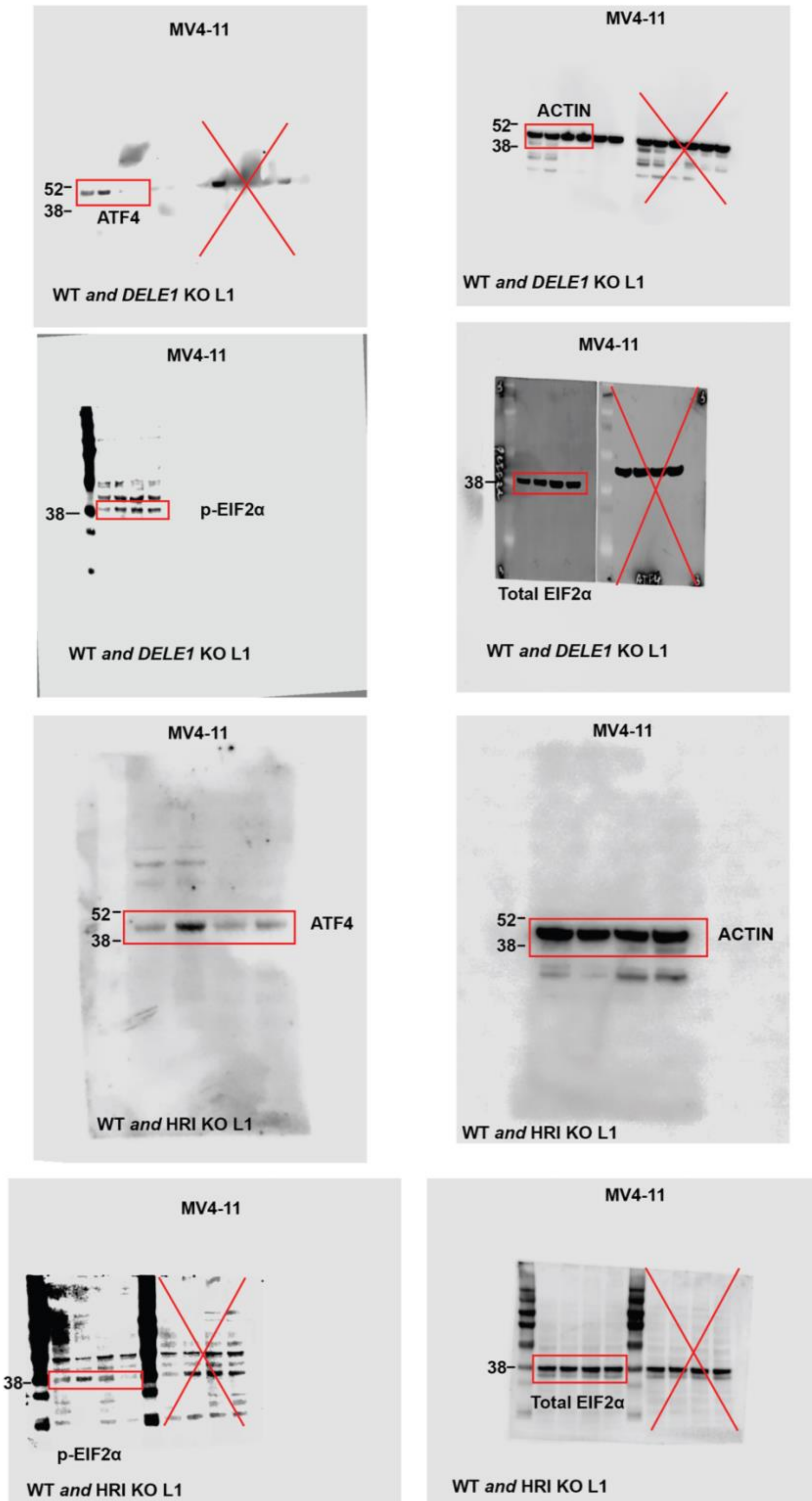

Supplementary Fig. S15D: Uncropped western blots for figure 4K.

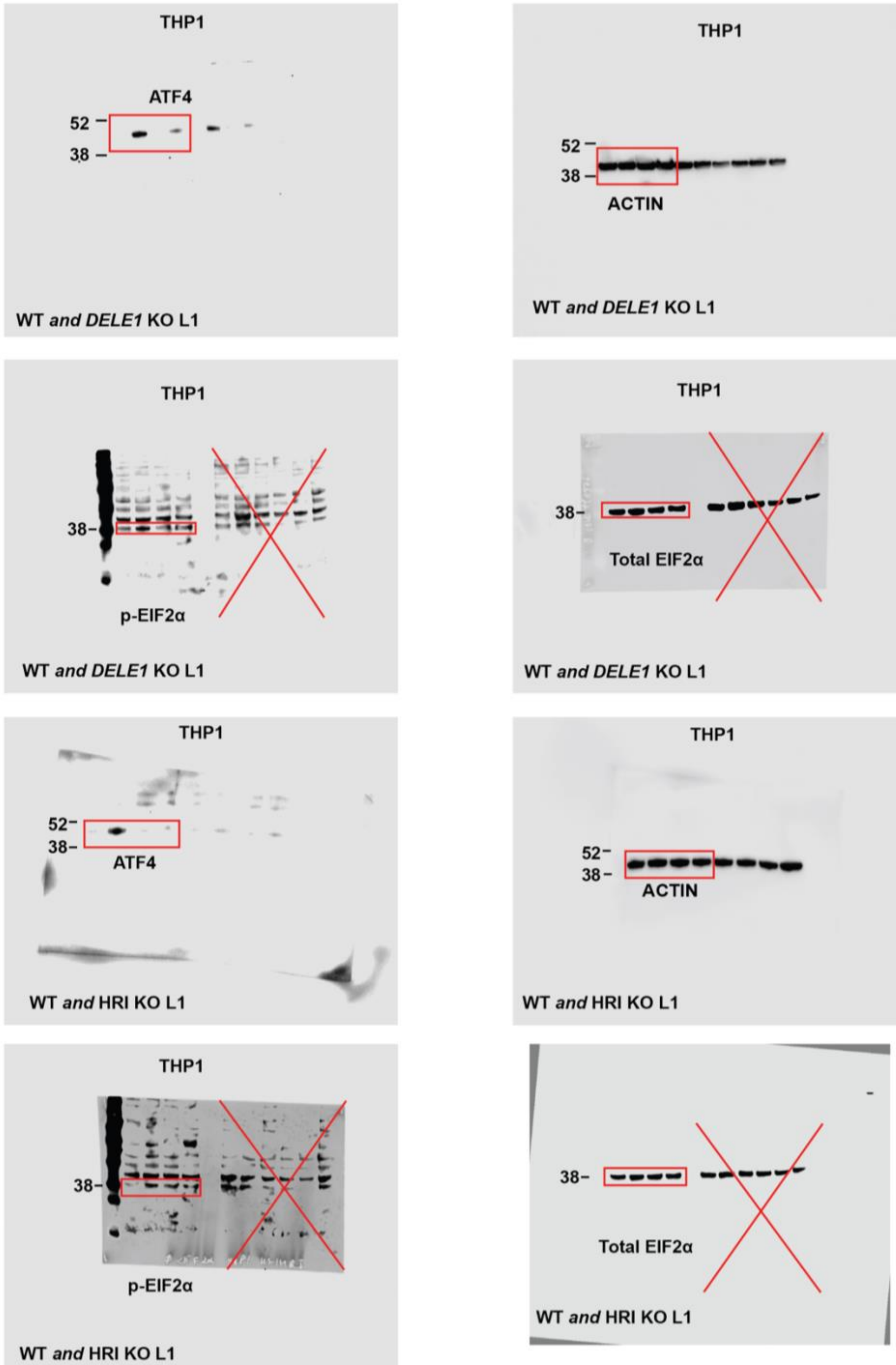

Supplementary Fig. S15E: Uncropped western blots for figure 4L.

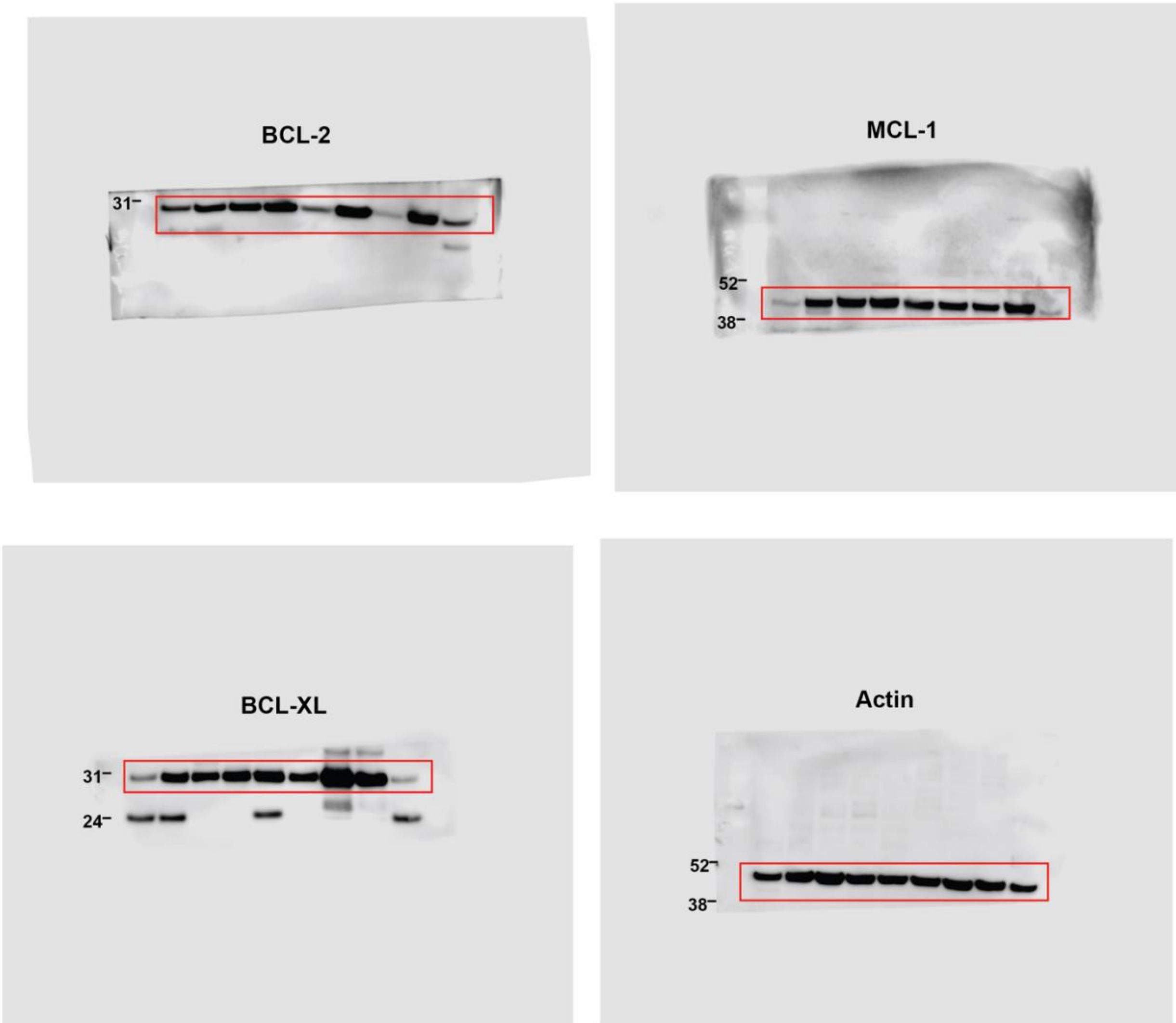

Supplementary Fig. S15F : Uncropped western blots for Supplementary Fig. S1.

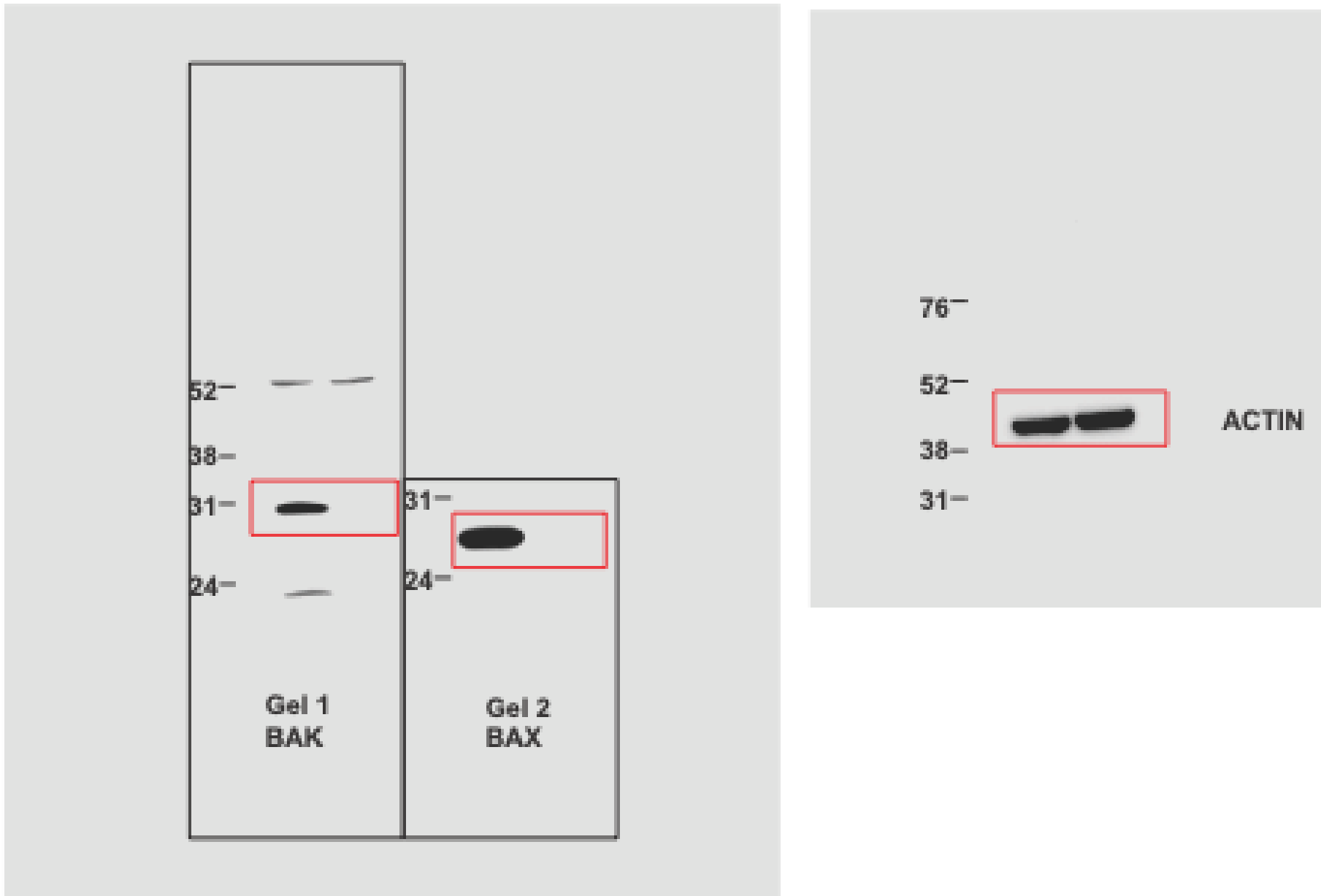

Supplementary Fig. S15G: Uncropped western blots for Supplementary Fig. S8A.

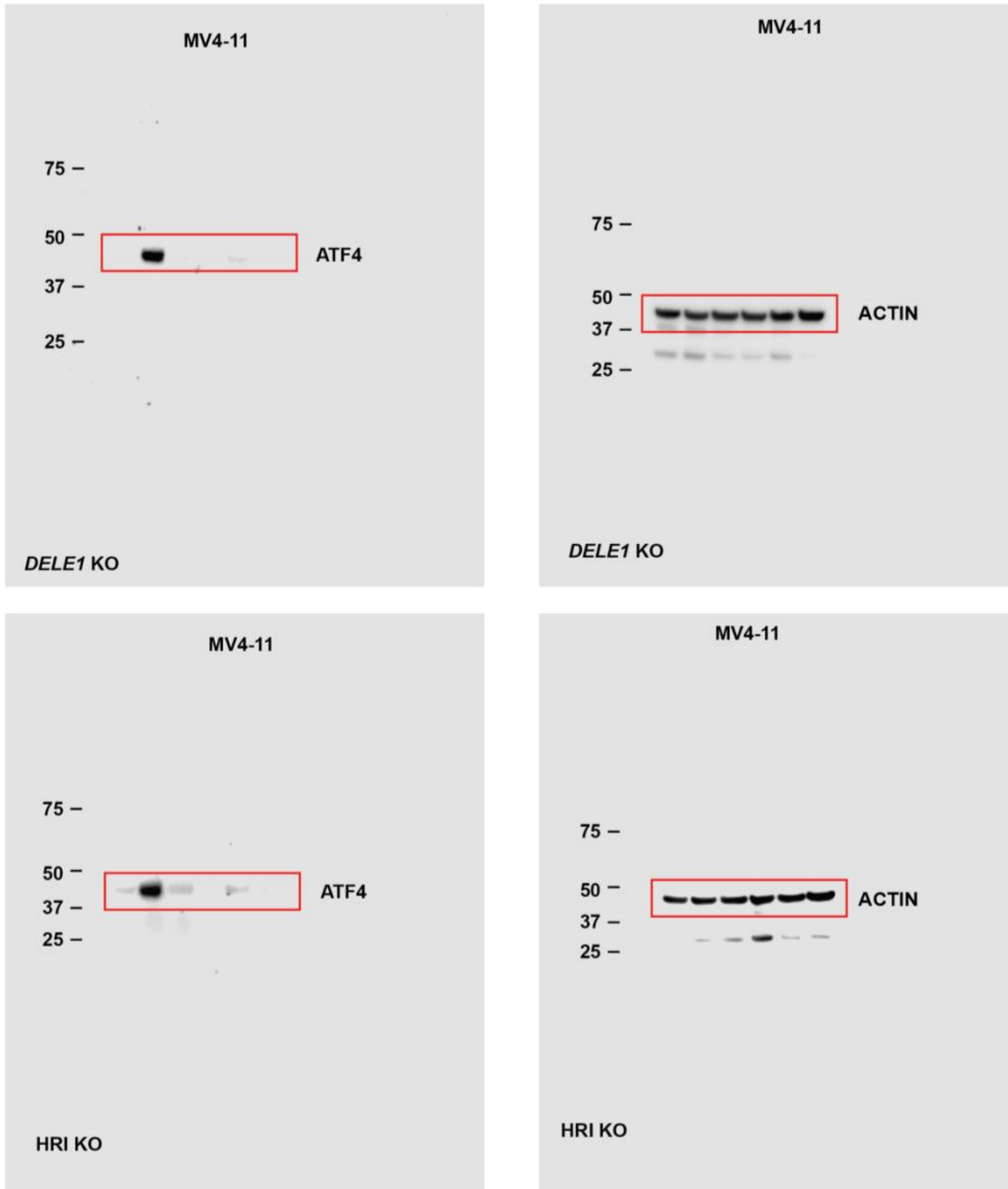

Supplementary Fig. S15H: Uncropped western blots for Supplementary Fig. 11A.

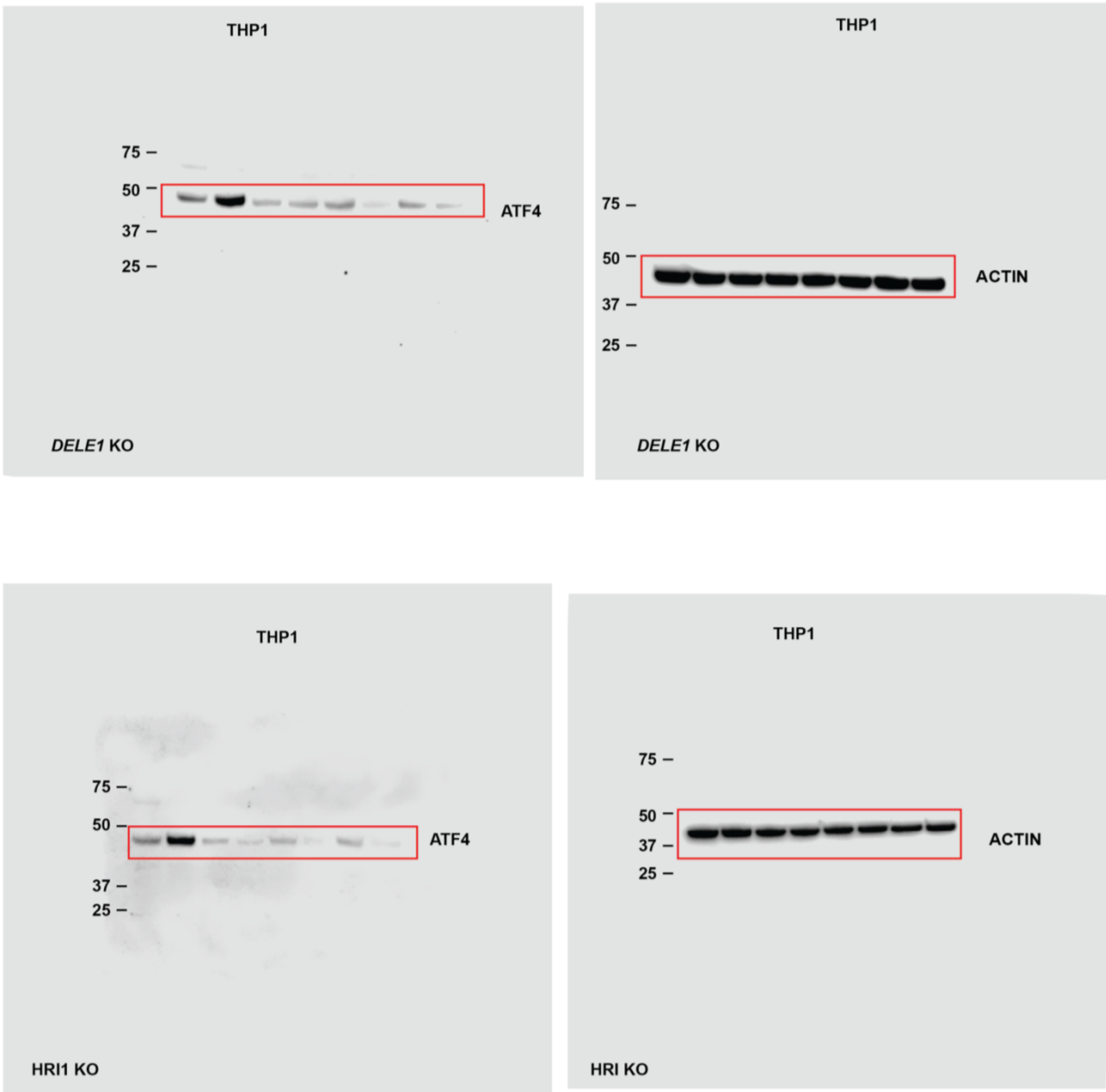

Supplementary Fig. S15I: Uncropped western blots for Supplementary Fig. 11B.

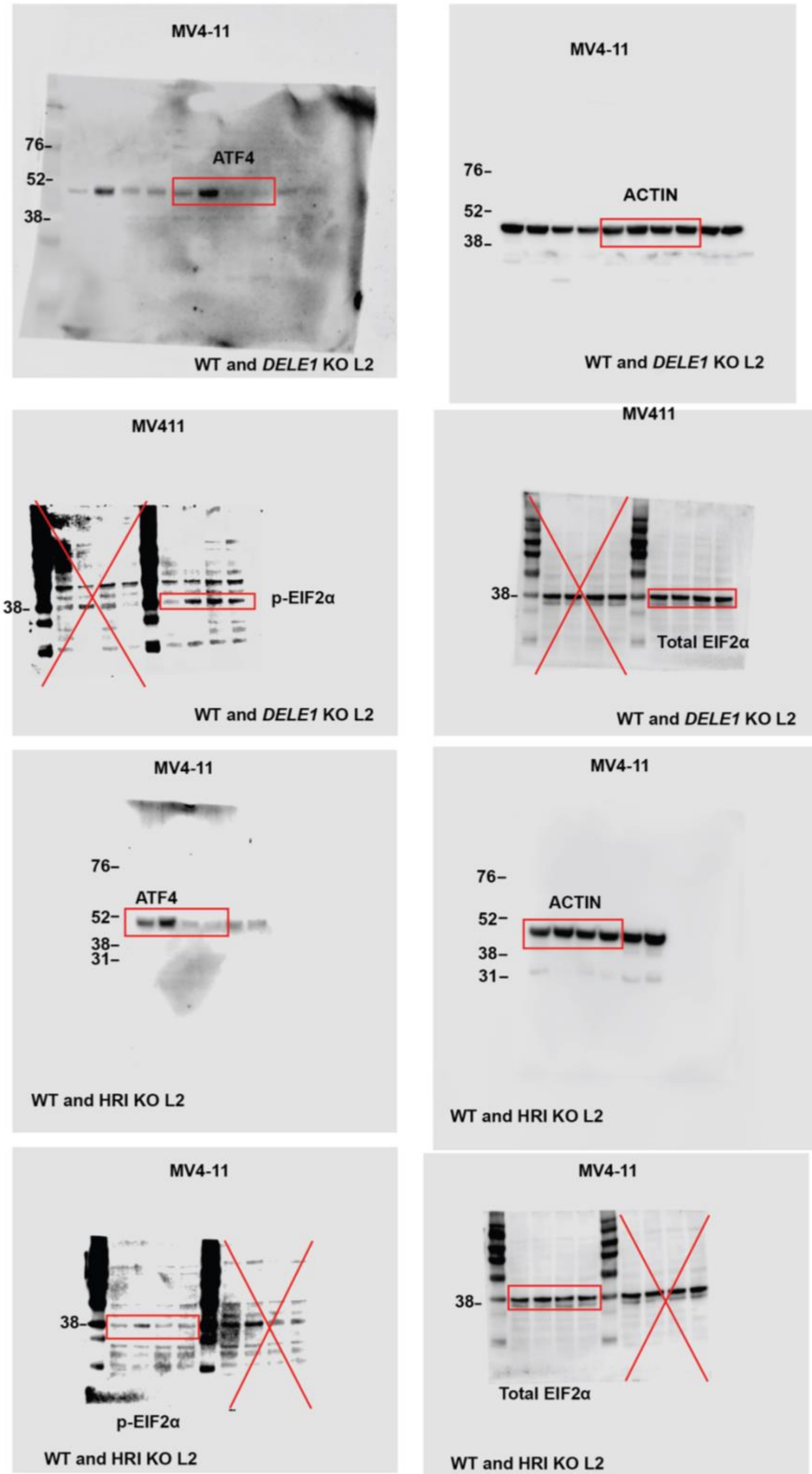

Supplementary Fig. S15J: Uncropped western blots for Supplementary Fig. 14I.

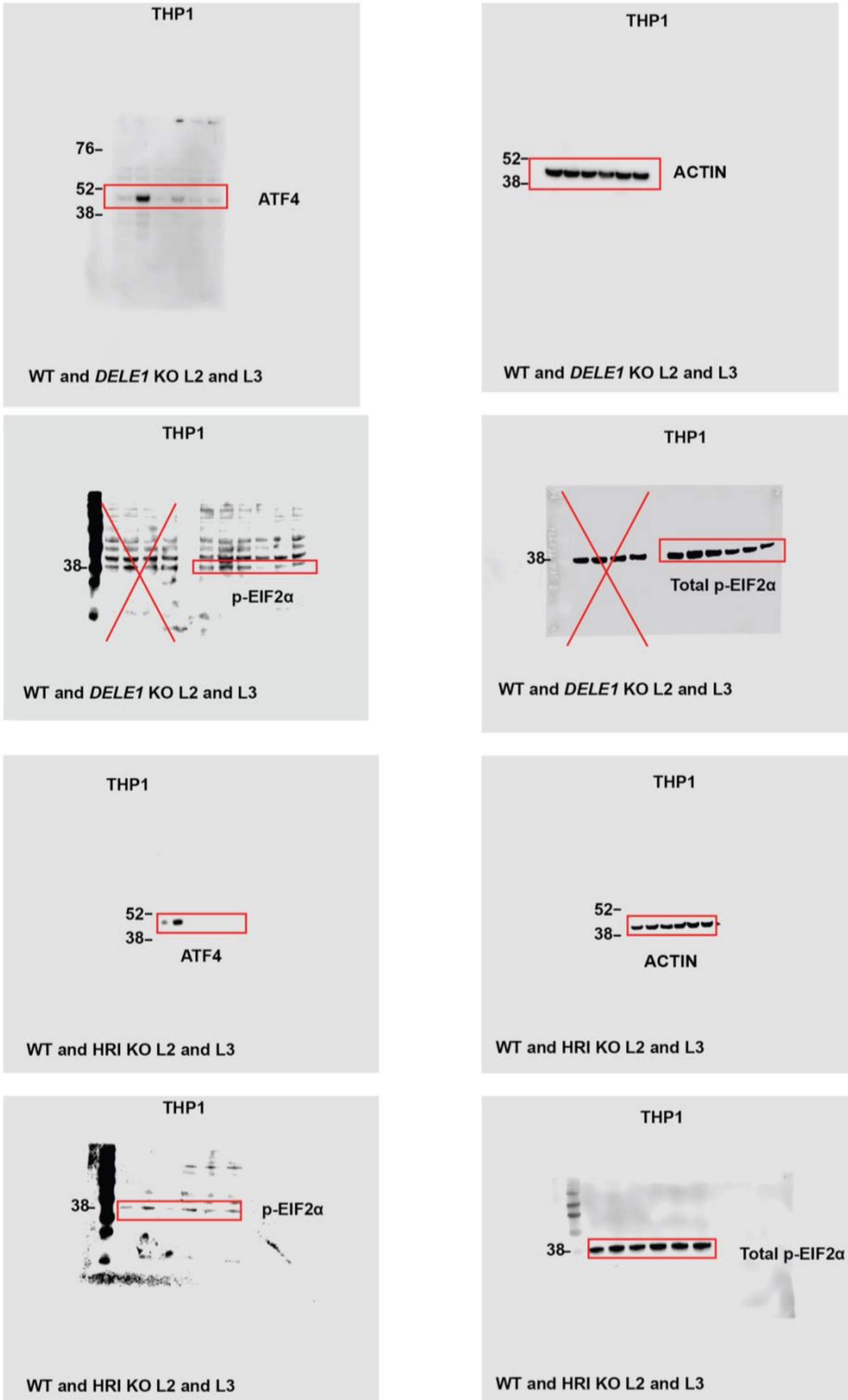

Supplementary Fig. S15K: Uncropped western blots for Supplementary Fig. 14J.
