# Supplementary material for: Mitochondrial integrated stress response activation creates a therapeutic vulnerability to MCL-1 inhibition in acute myeloid leukemia
Source: Cell Death Dis. 2026 Jun 25;17(1):785. doi: 10.1038/s41419-026-09037-w (PMC13558565; doi:10.1038/s41419-026-09037-w)
Supplement: Supplementary file 2 [file 41419_2026_9037_MOESM19_ESM.docx]

**Supplementary (S) Figures**

**Figure S1. Protein expression levels of the anti-apoptotic BCL-2 family members across a panel of acute myeloid leukemia cell lines.** Whole-cell lysates from indicated acute myeloid leukemia cell lines, cultured under basal cell culture conditions, were resolved by SDS-PAGE and protein expression was determined by immunoblot analyses to assess the endogenous protein expression of BCL-2, BCL-XL, and MCL-1. ACTIN was used as a loading control. Blots shown are representative of three independent experiments.

**Figure S2. *KMT2A*-r acute myeloid leukemia is sensitive to MCL-1 inhibition**. Cell viability of a panel of acute myeloid leukemia cells lines following 24 h treatment with vehicle (DMSO), A-1331852 (BCL-XL inhibitor), S63845 (MCL-1 inhibitor; tool compound for S64315), ABT-263 (BCL-2, BCL-XL, and BCL-W inhibitor), or ABT-199 (BCL-2 inhibitor) stained with APC-conjugated Annexin V (AV) and propidium iodide (PI). Viability (AV^-^/PI^-^) was normalized to the vehicle-treated (DMSO), control defined as 100% and represented as the symbols plotted on the y-axis. Data are plotted on a logarithmic scale with the drug concentration shown in nM. Values represent the mean of three or more experiments, with error bars representing the SEM.

**Figure S3. Representative flow cytometry plots of acute myeloid leukemia cells treated with DMSO or the indicated BH3 mimetics for 24 h at the approximate IC_50_ dose from Table 1**. Cells were stained with APC-conjugated Annexin V (AV) and propidium iodide (PI) to assess cell death. Plots display AV (APC) on the x-axis and PI on the y-axis, enabling discrimination of viable cells (AV^-^/PI^-^) from dead cells (AV^+^, PI^+^, and AV^+^/PI^+^). Plots from independent experiments dosed around the IC_50_ are shown.

**Figure S4. Dose optimization of the Metabolic CRISPR Knockout screen**. Cell viability of MV4-11 cells treated with indicated doses of AMG 176 **(A)** or S64315 **(B)** for 24 h. After the 24 h, drugs were removed from the media and cell viability was assessed 48 h post drug treatment assessed by staining with APC-conjugated Annexin V (AV) and propidium iodide (PI). These data were used to identify the dose that induced approximately 25% cell death for CRISPR screen in Fig. 1. Data are an average of three or more experiments, and error bars represent the SEM.

**Figure S5. Raw Seahorse Mito Stress Test data related to the Calculated ATP production graphs in Figure 2**. Representative oxygen consumption rate (OCR) graphs for ML2 **(A)**, MV4-11 **(B)**, and THP1 **(C)** cells treated with DMSO or 50 nM IACS for 1 h before the Seahorse Mito Stress Test under basal conditions and following sequential exposure to oligomycin (Oligo), carbonyl cyanide 4-(trifluoromethoxy) phenylhydrazone (FCCP), and rotenone/antimycin A (Rot/AA). Graphs are representative of three or more independent experiments and error bars represent the SEM. **D,** Both diagnosis and relapse patient-derived xenograft samples treated with DMSO or 50 nM IACS-010759 for 1 h prior to undergoing the Seahorse Mito Stress Test under basal conditions and following sequential exposure to Oligo, FCCP, and Rot/AA. Data are representative of four mice and error bars represent the SEM.

**Figure S6. MCL-1 inhibitors modestly repress OXPHOS**. **A,** Cell viability of acute myeloid leukemia cells treated with the indicated doses of S64315 after 24 h. Cell viability is normalized to the DMSO control, defined as 100%. Data are representative of three or more experiments and error bars represent the SEM. Oxygen consumption rate (OCR) graphs of MV4-11 **(B)**, MV4-11 DKO **(C)**, ML2 **(D)** , and THP1 **(E)** cells treated with the indicated doses of S64315 for 24 h prior to the Seahorse Mito Stress Test under basal conditions and following sequential exposure to oligomycin (Oligo), carbonyl cyanide 4-(trifluoromethoxy) phenylhydrazone (FCCP), and rotenone/antimycin A (Rot/AA). Data are representative of three or more experiments and error bars represent the SEM. Calculated ATP production graphs of MV4-11 **(F)**, MV4-11 DKO **(G)**, ML2 **(H)**, and THP1 **(I)** cells treated with the indicated doses of S64315 for 24 h prior quantified from the Seahorse Mito Stress Test. The values shown on the charts reflect the calculated percentage of ATP produced by the mitochondria (mitoATP) versus glycolysis (glycoATP), not direct ATP measurements. Data are combined from n=3 or more experiments and error bars represent the SEM.

**Figure S7. MCL-1 inhibitors synergize with IACS-010759.** Cell viability (AV^-^/PI^-^) of ML2 (**A-D)** and THP1 **(F-I)** cells treated with the indicated doses of IACS-010759 (IACS) combined with indicated doses of AMG 176 **(A,F)**, AZD5991 **(B,G)**, S63845 **(C,H)** and S64315 **(D,I)**. Cell viability is normalized to the DMSO-treated (Vehicle) control, defined as 100%. Graphs represent data from three or more independent experiments and error bars represent the SEM. Bliss synergy scores of ML2 **(E)** and THP1 **(J)** treated with IACS and AZD5991, AMG 176, S63845, or S64315. The dashed line indicates values over 10 as synergistic. P-values associated with Bliss synergy scores are calculated through SynergyFinder+ software. P-values < 0.0001 are indicated by ****.

**Figure S8. IACS-010759 + MCL-1i fail to induce apoptosis in the absence of BAX and BAK**. **A,** Immunoblot analysis of protein expression levels of BAK and BAX in *BAK*/*BAX* double knockout (DKO) and wild-type (WT) MV4-11 cells. ACTIN serves as loading control. **B,** Calculated ATP production graphs of WT and DKO MV4-11 cells treated with DMSO or 50 nM IACS-010759 (IACS) for 1 h before undergoing the Seahorse Mito Stress Test. The values shown on pie charts reflect the calculated percentage of ATP produced by the mitochondria (mitoATP) versus glycolysis (glycoATP), not direct ATP measurements. **C,** Raw oxygen consumption rates (OCR) data from the Seahorse Mito Stress Test under basal conditions and following sequential exposure to oligomycin (Oligo), carbonyl cyanide 4-(trifluoromethoxy) phenylhydrazone (FCCP), and rotenone/antimycin A (Rot/AA) from assay quantified in **S8B**. Seahorse data shown is representative of three experiments. **D-F,** Cell viability (AV^-^/PI^-^) of DKO cells treated with IACS combined with indicated doses of S64315, AMG 176 or AZD5991 for 24 h. Graphs represent data from three or more independent experiments, and error bars represent the SEM. Data is normalized to DMSO (vehicle) control, defined as 100%.

**Figure S9. Enrichr analysis from RNA sequencing of acute myeloid leukemia cell lines treated with IACS-010759.** MV4-11 **(A,C)** and THP1 **(B,D)** cells treated with IACS-010759 (IACS) for 8 h (**A, B**) or 24 h (**C, D**). For each cell line, the top differentially expressed transcripts were selected to identify the most significantly enriched pathways induced by IACS treatment for each timepoint. Differential expression was defined using threshold of p-value < 0.05 and |log2R| > 0.585 (corresponding to a half-fold change). The transcripts were analyzed using Enrichr to determine biological processes associated with IACS treatment.

**Figure S10. Representative raw oxygen consumption rate data from the Seahorse Mito Stress Test of the MV4-11 *ATF4* knockout (KO) cell line shown in Figure 3L**. Oxygen consumption rate (OCR) of *ATF4* KO MV4-11 cells pretreated for 1 h DMSO or 50 nM IACS-010759 (IACS) treatment were measured in real time under basal conditions and following sequential exposure to oligomycin (Oligo), carbonyl cyanide 4-(trifluoromethoxy) phenylhydrazone (FCCP), and rotenone/antimycin A (Rot/AA). The graph shown is representative of three experiments with at least 3 technical replicates per experiment. Error bars represent the SEM.

**Figure S11. Functional validation of DELE1 and HRI knockout.** **A and B,** All MV4-11 (**A**) and THP1 (**B**) DELE1 and HRI knockout (KO) cell lines treated with 10 μM carbonyl cyanide 4-(trifluoromethoxy) phenylhydrazone (FCCP) for 3 h and evaluated for ATF4 expression as determined by immunoblot analyses for indicated proteins. ACTIN serves as the loading control. Two to three independent enriched KO cell lines (L) per group were tested. Enriched cell line 1 (L1) from each panel serves as the representative cell line in the primary figures. L2 and L3 are presented only in supplementary figures.

**Figure S12. Loss of DELE1 or HRI does not alter response to IACS-010759**. **A and B,** Calculated ATP production of MV4-11 **(A)** and THP1 **(B)** WT, *DELE1*, and HRI KO lines treated with DMSO or IACS-010759 (IACS) for 1 h. The values shown on pie charts reflect the calculated percentage of ATP produced by the mitochondria (mitoATP) versus glycolysis (glycoATP), not direct ATP measurements. Two to three independent enriched KO cell lines (L) per group were tested. Enriched cell line 1 (L1) from each panel serves as the representative cell line in the primary figures. L2 and L3 are presented only in supplementary figures.

**Figure S13.** **Raw oxygen consumption rate data from the Seahorse Mito Stress Test presented in Figure S12**. All MV4-11 (**A-D**) and THP1 (**E-J**) DELE1 and HRI knockout (KO) cell lines treated with DMSO or IACS-010759 (IACS) for 1 h before the Seahorse Mito Stress Test was performed. Graphs shown are oxygen consumption rate **(**OCR) data from a representative of three experiments and error bars represent the SEM. Two to three independent enriched KO cell lines (L) per group were tested. The independent KO cell line 1 (L1) from each panel serves as the representative cell line in the primary figures. KO L2 and L3 are presented only in supplementary figures.

**Figure S14. Loss of *DELE1* and HRI promote resistance to IACS-010759 (IACS) and MCL-1 inhibition in multiple knockout-enriched cell lines.** Cell viability (AV^-^/PI^-^) of MV4-11 DELE1 L2 **(A)** and HRI knockout (KO) L2 **(B)** cell lines treated with IACS and S64315 for 24 h. Viability is normalized to a DMSO (vehicle) control, defined as 100%. **C,** Bliss synergy scores of the additional *DELE1* and HRI KO cell lines. The dashed line indicates values >10 as synergistic. Statistical analysis is showing Zp-value comparing Bliss synergy scores and p-values between the WT (wild-type, Bliss synergy score shown in Figure 4J) and additional KO cell lines. Zp-values < 0.01 are indicated by ** and < 0.0001 are indicated by ****. THP1 *DELE1* L2 and L3 **(D and E)** and HRI KO L2 and L3 **(F and G)** cell lines treated with IACS and S64315 for 24 h. **H,** Bliss synergy scores of the THP1 *DELE1* and HRI KO cell lines. The dashed line indicates values over 10 as synergistic. Statistical analysis is showing Zp-value comparing Bliss scores and p-values between the WT (Bliss synergy score shown in Figure 4J) and additional KO cell lines. Zp-values < 0.0001 indicated by ****. Graphs represent data from three or more independent experiments, and error bars represent the SEM. **I and J,** Western blot analysis of p-EIF2⍺ and ATF4 activation in the MV4-11 **(I)** and THP1 **(J)** additional KO cell lines treated with IACS. Independent KO cell lines (L) labeled as L2 and L3 are presented only in supplementary figures.

**Figure S15. Uncropped Western blots.** Full-length images of representative Western blots from all figures. Un-cropped blots are annotated with relevant molecular weight markers, antibodies used, cell lines, and genotypes. Blots in the uncropped image, but not included in the paper are marked with an “X.” Regions corresponding to the bands shown in the figures are highlighted using a red box. Images are representative of independent experiments.
