# Supplementary material for: Mitochondrial integrated stress response activation creates a therapeutic vulnerability to MCL-1 inhibition in acute myeloid leukemia
Source: Cell Death Dis. 2026 Jun 25;17(1):785. doi: 10.1038/s41419-026-09037-w (PMC13558565; doi:10.1038/s41419-026-09037-w)
Supplement: Supplementary file 4 [file 41419_2026_9037_MOESM2_ESM.docx]

**Table S2: Primer and probe set sequences used to determine viral titer.** Sequences of the primers and probe sets used to determine lentiviral titer by droplet digital PCR.

| **Target** | **Primer** | **Probe** |
| --- | --- | --- |
| **HIV Psi** | 5’-ACTTGAAAGCGAAAGGGAAAC-3’  5’ -CAC CCATCTCTCTCCTTCTAGCC- 3’ | 5’-56-FAM-AGCTCTCTC-ZEN-GACGCAGGACTCGGC-3IABkFQ-30 |
| **RPP30** | 5’ -GCGGCT GTCTCCACAAGT-3’  5’ -GATTTGGACCTGCGAGCG-3’ | 5’-5HEX-CTGACCTGA-ZEN-AGGCTCT-3IABkFQ-3’ |
