# Supplementary material for: Mitochondrial integrated stress response activation creates a therapeutic vulnerability to MCL-1 inhibition in acute myeloid leukemia
Source: Cell Death Dis. 2026 Jun 25;17(1):785. doi: 10.1038/s41419-026-09037-w (PMC13558565; doi:10.1038/s41419-026-09037-w)
Supplement: Supplementary file 5 [file 41419_2026_9037_MOESM3_ESM.docx]

**Table S3: CRISPR-Cas9 editing construct sequences.** Sequences of the single guide RNA (sgRNA) used to knockout the human genes indicated in the THP1 and/or MV4-11 cell lines. Primer sequences used for next generation sequencing (NGS) to detect the presence of gene insertions and/or deletions.

| **Name** | **Sequence (5’ to 3’)** |
| --- | --- |
| **sgRNA spacer sequence** | |
| CAGE2466.ATF4.g1 | UCAGAACAGCUAACCUCUAA |
| CAGE2470.ATF4.g4 | UGGGUGGGUGGCGCUUCACU |
| CAGE2468.DELE1.g2 | ACUGGGACCUAGCCUCUGGA |
| CAGE410.EIF2AK1.g4 | UUGUUGGCUAUCACACCGCG |
| CAGE237.BAK.g2 | GCUCACCUGCUAGGUUGCAG |
| CAGE357.BAX.g1 | CUGCAGGAUGAUUGCCGCCG |
| **NGS primer sequences (NGS overhangs in upper case)** | |
| CAGE2466.ATF4.DS.F | CTACACGACGCTCTTCCGATCTcccctggtctccgtgagcgtccatt |
| CAGE2470.ATF4.DS.R | CAGACGTGTGCTCTTCCGATCTgccaactatacggctccagggcc |
| CAGE2468.DELE1.DS.F | CTACACGACGCTCTTCCGATCTacgtggatgctctaccctgagaaatc |
| CAGE2468.DELE1.DS.R | CAGACGTGTGCTCTTCCGATCTtgggcgacagaatgagactccatct |
| CAGE410.EIF2AK1.DS.F | CTACACGACGCTCTTCCGATCTaggggccatctgtattttgctctgga |
| CAGE410.EIF2AK1.DS.R | CAGACGTGTGCTCTTCCGATCTaatggtgtgaacccaggaggcggag |
| CAGE237.BAK.DS.F | CTACACGACGCTCTTCCGATCTtgactcccagctttgatcctcagagg |
| CAGE237.BAK.DS.R | CAGACGTGTGCTCTTCCGATCTgagcccaacataaaagaggagcaacc |
| CAGE357.BAX.DS.F | CTACACGACGCTCTTCCGATCTttcccttgtcccccgttggcctgtt |
| CAGE357.BAX.DS.R | CAGACGTGTGCTCTTCCGATCTctgcagctgcccaccttgagcacc |
